# Snf7 spirals sense and alter membrane curvature

**DOI:** 10.1038/s41467-022-29850-z

**Published:** 2022-04-21

**Authors:** Nebojsa Jukic, Alma P. Perrino, Frédéric Humbert, Aurélien Roux, Simon Scheuring

**Affiliations:** 1 Physiology, Biophysics and Systems Biology Graduate Program, Weill Cornell Medicine, New York, NY 10065 USA; 2 Department of Anesthesiology, Weill Cornell Medicine, New York, NY 10065 USA; 3 Department of Biochemistry, University of Geneva, CH-1211 Geneva, Switzerland; 4 Swiss National Centre for Competence in Research Programme Chemical Biology, CH-1211 Geneva, Switzerland; 5 Department of Physiology and Biophysics, Weill Cornell Medicine, New York, NY 10065 USA; 6 Kavli Institute at Cornell for Nanoscale Science, Cornell University, Ithaca, New York, NY 14853 USA

**Keywords:** Membrane biophysics, ESCRT, Molecular biophysics

## Abstract

Endosomal Sorting Complex Required for Transport III (ESCRT-III) is a conserved protein system involved in many cellular processes resulting in membrane deformation and scission, topologically away from the cytoplasm. However, little is known about the transition of the planar membrane-associated protein assembly into a 3D structure. High-speed atomic force microscopy (HS-AFM) provided insights into assembly, structural dynamics and turnover of Snf7, the major ESCRT-III component, on planar supported lipid bilayers. Here, we develop HS-AFM experiments that remove the constraints of membrane planarity, crowdedness, and support rigidity. On non-planar membranes, Snf7 monomers are curvature insensitive, but Snf7-spirals selectively adapt their conformation to membrane geometry. In a non-crowded system, Snf7-spirals reach a critical radius, and remodel to minimize internal stress. On non-rigid supports, Snf7-spirals compact and buckle, deforming the underlying bilayer. These experiments provide direct evidence that Snf7 is sufficient to mediate topological transitions, in agreement with the loaded spiral spring model.

## Abstract (web summary)

Snf7 is the major component of the ESCRTIII membrane deformation system. Here, the authors used high-speed AFM to study Snf7 on nano-patterned and soft supports and show that loaded Snf7 spiral springs are curvature sensitive and deform membranes.

## Introduction

Cells use various mechanisms to remodel the shape of its constituent membranes, i.e. generate curvature, such as heterogeneity in the lipid composition^1,2^, self-assembly or insertion of proteins^3^, and cytoskeletal rearrangements^4,5^. The Endosomal Sorting Complex Required for Transport III (ESCRT-III), a complex of the larger ESCRT system, are peripheral membrane proteins that transiently interact with membranes, each other and with other ESCRT-associated proteins. They are crucial in processes where the membrane is budded away from the cytoplasm and the budding neck thus formed is constricted and severed - first described in the formation of multi-vesicular bodies in yeast^6–8^. They are implicated in a wide range of physiological and pathophysiological roles. The ESCRT pathway is hijacked by nascent encapsulated virions (such as HIV-1) in order to fission from infected cells^9–12^. The ESCRT system is evolutionarily well conserved in *Eukaryota*, and ESCRT-III homologs are also found in certain *Archaea*, where they play a key role in cell division^13–15^. Thus, the presence of a functional ESCRT system is of importance to the normal functioning of cells.

Since the ESCRT system was first discovered, there has been a continued focus on understanding the mechanistic details of ESCRT-III function. First, electron microscopy observations of ESCRT-III components provided images of the architecture of ESCRT-III assemblies in vitro^16^ and in vivo^17^. CHMP2 and CHMP3 formed helical assemblies on the outside of a membrane tubule^16^. Upon overexpression of CHMP4 only and co-expression of CHMP4 with hydrolysis-defective Vps4, flat spirals and tubular plasma membrane evaginations containing a protein helix have been imaged, respectively^17^. Similarly, cryo-EM studies of *Caenorhabditis elegans* Vps32, homologous to CHMP4B, showed that this protein formed single-filament spirals^18^. Based on these observations, models have been proposed regarding the ability of ESCRT-III to induce membrane scission, a process requiring sequential recruitment of several ESCRT-III components (Snf7, Vps2, Vps24, canonically being considered the core components) and the ESCRT-III associated ATPase Vps4^19–25^. However, these models rarely addressed membrane budding, which was at times proposed to be due to the action of upstream ESCRT components^26,27^ or to a decrease in membrane tension resulting in membrane undulation^28,29^. Nevertheless, the observation of planar spiral structures of CHMP4B orthologs led to the hypothesis that these structures could accumulate potential energy because of the deviation from a preferred bending curvature of the filaments^18^. This was further substantiated by high-speed atomic force microscopy observations of spiral structures of yeast Snf7 (homologous to CHMP4B) on supported lipid bilayers^30^. On the other hand, electron microscopy studies of Snf7 overexpressing cells revealed concave-conical Snf7 spirals capping tubular protrusions on the inner leaflet^17,31^, thus providing observations of both planar and non-planar higher-order structures of Snf7. This, in combination with reports of i) Snf7 being the most abundant ESCRT-III component (~150 nM concentration in yeast cells)^32–35^, and ii) mechanistic details governing Snf7 conformational changes, loss of autoinhibition and subsequent polymerization^36–39^, made Snf7 a prime candidate for a molecular machinery that is able to assemble on lipid bilayers and initiate membrane budding. In this context, fluorescence microscopy of GUVs in the presence of yeast ESCRT-III components demonstrated that Snf7 alone was sufficient for membrane budding^40^, although with low efficiency, and unable to induce membrane scission.

Taken together, these observations led to the proposal of a loaded spiral spring model^41^, predicting that Snf7 monomers polymerize into filaments on a flat membrane, and assuming that these filaments had a preferred radius of curvature. According to this model, as the filaments polymerize further into spirals, more distal spiral turns must adapt to lesser and lesser curvature, thus applying a force towards the spiral center, where the inner turns become over-curved. Thus, both the outer and inner turns represent high-energy states, with energy being accumulated through polymerization. The spiral eventually collapses into a helix, where all filament turns adopt their preferred radius of curvature. This reductionist model does not consider the role of upstream ESCRT components that could provide crucial signals regulating the spatial and temporal localization of Snf7 assemblies in vivo or in reconstituted systems in vitro. At its simplest, the model also ignores the role of downstream ESCRT-III components that could remodel Snf7 assemblies to further change the geometry of the underlying membrane. A more recent development of the model proposes that membrane deformation to ever more constricted tubular structures was achieved through a Vps4-mediated, ATP-driven turnover of various ESCRT-III components^23,24,42^. A crucial step in this model is the initial membrane deformation, due to release of stress accrued by a mismatch of the filament-membrane binding angle on a planar membrane as proposed by in silico modeling^42,43^, summarized in^44,45^. However, all models rely on spontaneous spiral buckling, which has never been observed at the molecular scale.

Supported lipid bilayers (SLBs) have successfully been used as an experimental model system to investigate membrane phenomena in HS-AFM studies of Snf7 assembly and dynamics^24,30^. However, HS-AFM experiments using traditional supports precluded from elucidating the mechanism by which Snf7 could deform the underlying membrane because several constraints were imposed on the Snf7-lipid bilayer system. First, these supports are planar, whereas lipid membranes in cells display a variety of curvatures, and thus planar SLBs do not reflect faithfully the membrane geometry that Snf7 encounters in living cells (Supplementary Fig. 1). Second, traditional AFM supports (*e.g*. mica, glass, silicon) are rigid, and therefore prohibit any meaningful out-of-plane deformation. Third, in the former HS-AFM experiments, Snf7 crowding facilitated imaging but represented lateral constraints potentially obscuring structural and dynamical insights^24,30^.

Here, in order to observe Snf7 spirals in lower-energy, out-of-plane deformed states, we designed HS-AFM experiments in which we eliminated the abovementioned constraints, membrane planarity, support rigidity and Snf7 crowding. We find that Snf7 spirals can adapt to convex and concave curvatures, but cannot interchange the topological sign, indicative of a filament bending rigidity that exceeds the bending rigidity of the membrane. Snf7 spirals reach a critical radius of curvature limited by the energy state of the outer turns. Snf7 spirals alone can rearrange and transition from a flat spiral into a helical cone. Altogether, our findings provide direct evidence of the loaded spiral spring model.

## Results

### Non-planar supported lipid bilayers

Our first approach in understanding whether individual Snf7 assemblies could assume out-of-plane buckled conformations consisted of Snf7 being presented with membrane curvature of biologically relevant magnitudes. We reasoned that Snf7 assemblies, unable to remodel the membrane due to the flatness and rigidity of classical microscopy supports, could instead adapt to polymerize preferentially in geometrically favorable areas on an undulated surface, thus adopting a geometry closer to their energy minimum. A similar approach has been attempted in a previous study by tracking the dynamics of Snf7 polymerization on SLBs deposited on micron-sized pits in a rigid substrate^46^. In the aforementioned study, Snf7 reportedly polymerized preferentially in areas of the SLB presenting negative membrane curvature, yet the limitations of this approach were twofold: i) the resolution of the microscopy method used did not allow investigation of the geometry of individual Snf7 spirals, and ii) the areas on the rims of the pits displayed significant roughness in AFM, and while the surface could be interpreted to be negatively curved on the microscale, the nanoscale curvature could vary significantly. In addition, observation of Snf7 polymerization on non-planar surfaces should inform us about curvature sensitivity of Snf7 on the monomer and oligomer scales.

**Fig. 1:**
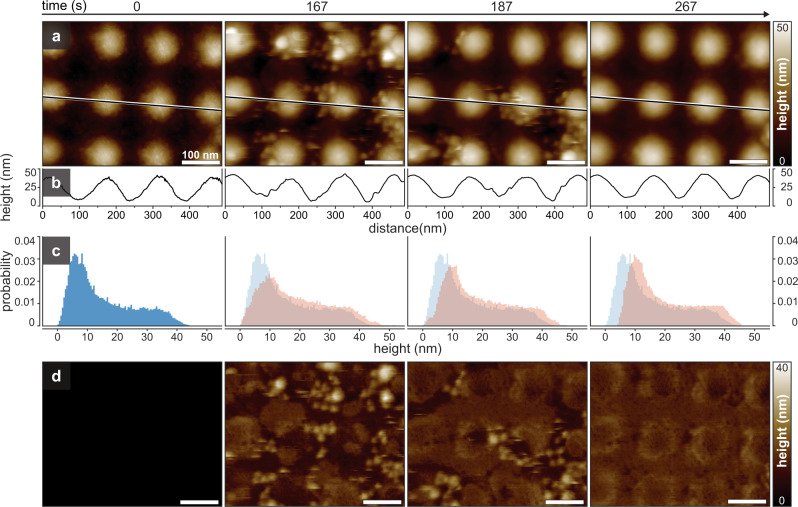
Time-lapse of SLB formation on undulated (concave-convex) nanopatterned HS-AFM supports. **a** HS-AFM frames (Supplementary Movie 1) of the bilayer formation process: *t* = 0 s: Non-flat sample surface before addition of lipid. *t* = 167 s: SUVs deposit on the surface. *t* = 187 s: Vesicles start to spread and fuse to form a SLB. *t* = 267 s: The entire surface is membrane covered. **b** Line profiles along the dashed lines in **a**. **c** Height distribution histograms of the images shown in **a**: Height values shifted over time by the thickness of a bilayer (~4 nm; compare blue and orange height distributions at *t* = 0 s and *t* = 267 s, respectively. **d** Nanopattern-subtracted images of the images shown in **a**. Source data are provided as a Source Data file.

In order to observe Snf7 membrane binding and polymerization on membranes displaying curvature, we used a support with a smoothly undulated surface of ~150 nm periodicity, ~35 nm amplitude and ~75 nm radius (see Methods and Supplementary Fig. 5) as an experimental platform (Fig. 1, *t* = 0 s). SLB formation is performed by supplementing the imaging solution in the HS-AFM fluid cell with small unilamellar vesicles (SUVs) of a DOPC/DOPS (6/4, mol:mol) mixture. Upon addition of the SUVs, individual vesicles adhering to the support surface were observed (Fig. 1, *t* = 167 s). Spreading of the vesicles led to the formation of lipid bilayer patches (Fig. 1, *t* = 187 s), that fused into a continuous SLB within minutes (Fig. 1, *t* = 267 s, Supplementary Movie 1). The bilayer formation was further confirmed by section (Fig. 1b) and height distribution analysis (Fig. 1c). Upon SLB formation all pixel height values increased by ~4-5 nm, when comparing the initial bare sample support (Fig. 1a, c, *t* = 0 s) to the final bilayer-covered surface (Fig. 1a, c, *t* = 267 s). Thus, this procedure allowed us to form SLBs with convex hills separated by saddle-point shaped valleys, with shallow concave interstices separating four neighboring protrusions (Fig. 1a, b, *t* = 267 s).

### Snf7 monomers are not membrane curvature sensitive

**Fig. 2:**
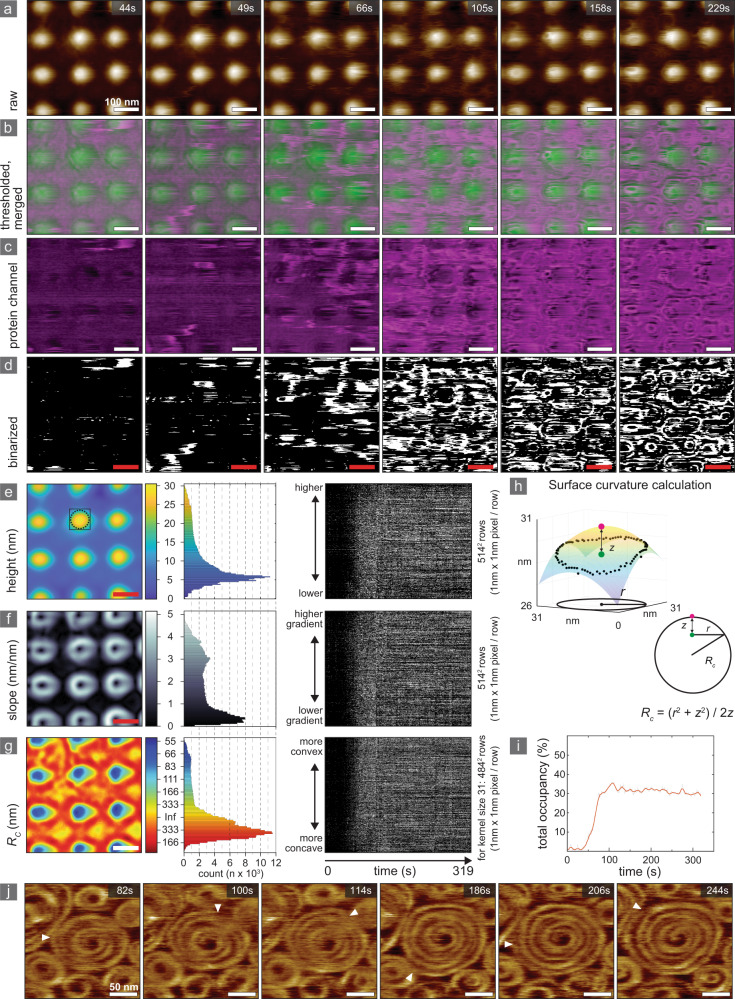
Snf7 adsorption and polymerization has no curvature preference. HS-AFM images (Supplementary Movie 2) showing the polymerization of Snf7 on an undulated (concave-convex) nanopatterned support covered with a SLB: Raw data HS-AFM frames (**a**), merged support-channel (green) and Snf7 protein-channel (magenta) (**b**), separate Snf7 protein-channel (magenta) (**c**) and binarized protein-channel (**d**). Left: Substrate height (**e**), slope (**f**), and curvature (**g**) maps calculated from the support-channel. Middle: Binned histograms of height (**e**), slope (**f**) and curvature (**g**), respectively, with bin area proportional to the number of pixels of a given characteristic. Right: Unbinned occupancy of individual pixels by Snf7 (**d**) as a function of time (horizontal axis) and sorted by surface characteristic height (**e**), slope (**f**) and curvature (**g**). Each row represents an individual pixel, and each column represents a timepoint. **h** Schematic of spherical cap fitting procedure for surface curvature calculation. A sphere is fit to a ring of pixels with a user-defined radius (*r*, in this case, 15 pixels) from the central pixel. The radius of the fitted sphere (*R*_*c*_) is determined from the ring radius (*r*) and the difference (*z*) between the mean height value of the pixels within the ring (green dot) and the height of the central pixel (magenta dot). The example shown in **h** corresponds to the outline in **e**. **i** Snf7 surface occupancy as a function of time, shown as percentage of the total frame pixel number. **j** HS-AFM visualization of spiral growth: the filament grows at the spiral periphery (Supplementary Movie 3). Source data are provided as a Source Data file.

Once the support surface was fully covered with a lipid bilayer, thus presenting membrane surfaces of varying prominence, slope and curvature, Snf7 was added to the imaging solution at a final concentration of ~3 μM (Fig. 2, Supplementary Movie 2, Supplementary Fig. 5). HS-AFM observation of Snf7 polymerization on non-planar bilayers was challenging due to the amplitude of the support features exceeding the height of Snf7 (Fig. 2a). We separated the raw HS-AFM movies into a substrate channel and a Snf7-channel using a custom thresholding algorithm (Supplementary Fig. 2 and Methods section). The substrate surface (support) channel was obtained by smoothing the raw data frames using a Gaussian filter (Fig. 2b, green). The Snf7-channel (Fig. 2b, c, magenta) was obtained by subtracting the support channel from the raw data. The custom thresholding algorithm utilized different threshold values for topographically different regions of the surface, allowing us to binarize the protein channel so that the structural features of Snf7 spirals could be resolved (Fig. 2d). The result of this binarization allowed us to quantify the Snf7 assembly process at each position and time point during the experiment (Fig. 2d). After an initial lag period, Snf7 adhered to the SLB and short filaments and small-diameter annular structures were detected (Fig. 2a–d, *t* = 44 s). Snf7 polymerization proceeded rapidly thereafter (as has been observed on planar surfaces^24,30^) reaching full coverage in just about one minute (Fig. 2a–d, *t* = 105 s), and fully formed spirals were resolved shortly after (Fig. 2a–d, *t* = 229 s).

The early polymerization stages were highly dynamic, and Snf7 assemblies displayed a high degree of diffusivity and structural remodeling. Assembly remodeling decreased abruptly once the surface of the sample was crowded with Snf7, and spiral assemblies that were established during this stage remained stable for long periods of time (Supplementary Movie 2).

In parallel to the Snf7 occupancy analysis in the protein channel, we analyzed the support-channel regarding height (Fig. 2e), slope (Fig. 2f) and curvature (Fig. 2g, h) at every pixel (see Methods section), and binned them according to their characteristics (Fig. 2e–g, middle). We then sorted the individual pixels based on the surface characteristics of the substrate channel before Snf7 addition and polymerization and plotted the Snf7 occupancy of each pixel as a function of time (Fig. 2e–g, right). We found that Snf7 surface occupancy was uniformly distributed across all surface support characteristics (≤105 s in Fig. 2e–g, middle). Polymerization completed from *t* = 44 s to *t* = 105, after which occupancy plateaued for the remainder of the experiment (Fig. 2i). From these analyses, we concluded that localization of individual Snf7 molecules and assemblies was unaffected by membrane height, slope, and, most importantly, curvature. On flat substrates, we were able to directly image the molecular growth process, and found that spirals grew at the periphery, with a maximum rate of 8.5 nm/s in crowded conditions (Fig. 2j, Supplementary Movie 3).

### Snf7 spirals are membrane curvature sensitive

An advantage of our approach was the periodic nature of the surface protrusions. We reasoned that, if there were a few favorable Snf7 assembly conformations, the distribution of spiral sizes and locations on the undulated surface should follow a multimodal distribution. Alternatively, if Snf7 spirals were extremely flexible and able to adopt many conformations, the spiral size and positioning distributions should be uniform.

**Fig. 3:**
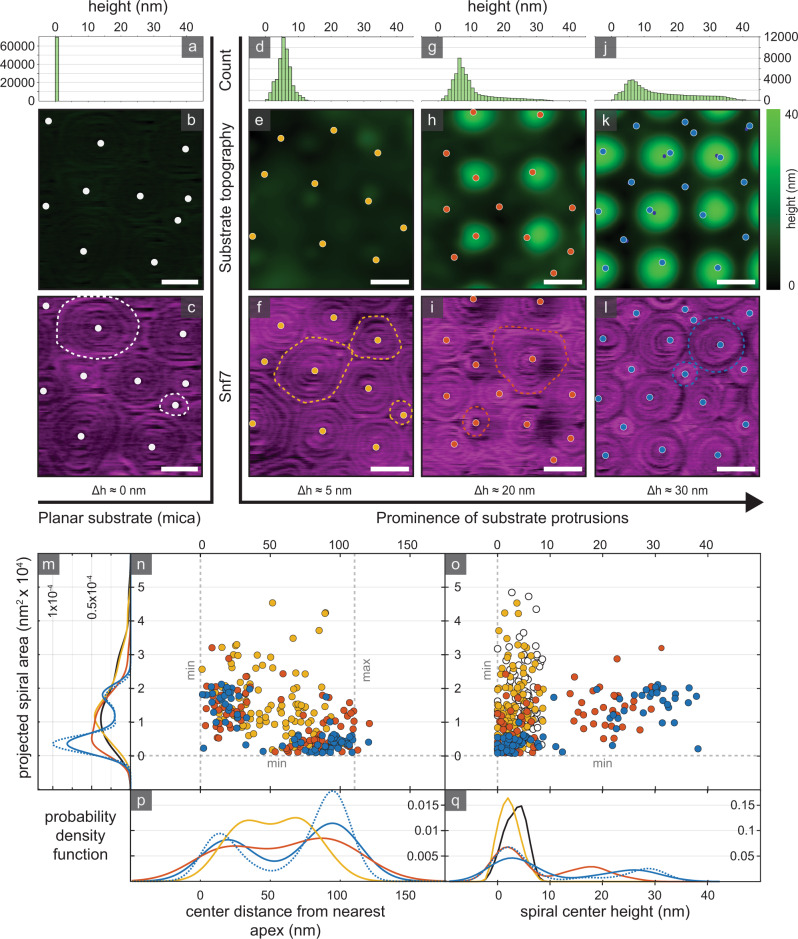
Snf7 spirals are membrane curvature sensitive. Height distributions of a planar support (**a**) and three different undulated supports (**d**, **g**, **j**). Support-channel of planar (**b**) and undulated supports of low (Δh ≈ 8 nm, **e**), moderate (Δh ≈ 25 nm, **h**), and prominent (Δh ≈ 35 nm, **k**) protrusions. (**c**, **f**, **i**, **l**) Protein-channel of Snf7 spirals on substrates (**b**, **e**, **h**, **k**), with representative spirals highlighted (dashed outlines). **m** Distributions of 2D-projected Snf7 spiral areas. Scatter plots of Snf7 spirals, with 2D-projected area versus spiral center distance from nearest apex (**n**) and versus spiral center height (**o**). Distributions of spiral center distance from nearest apex (**p**), and spiral center height (**q**). White: *n* = 99 spirals, 5 different imaging areas. Yellow: *n* = 98 spirals, 5 different imaging areas. Red: *n* = 80 spirals, 4 different imaging areas. Blue: *n* = 78 spirals, 5 different imaging areas. Scale bars: 100 nm. Plots (**m**–**q**) are shown separated by substrate in Supplementary Fig. 3. Source data are provided as a Source Data file.

Snf7 spirals that polymerized on planar SLBs had varying size (Fig. 3a–c). Size analysis revealed a long tail in the distribution corresponding to large spirals (Fig. 3m, o, black). Snf7 spirals on substrates with only minor surface protrusions (Fig. 3d–f), showed a similar size distribution to spirals on planar SLBs (Fig. 3m–o, yellow). On such faint protrusions, spiral centers rarely coincided with the apices (Fig. 3p, yellow). However, as the prominence of the substrate protrusions increased (Fig. 3g–i), the spiral size distribution narrowed (Fig. 3m–o, red), and an increased number of spiral centers coincided with the protrusion apices (Fig. 3p, red). Finally, in experiments with substrates featuring highly prominent protrusions (Fig. 3j–l), spirals segregated into two distinct populations: the spiral size (Fig. 3m–o, blue) and location (Fig. 3p, blue) distributions were bimodal. Spirals either centered at the apices of the convex protrusions (apical spirals), or centered in the concave areas nested between four adjacent apices (interstitial spirals). The spirals in these populations differed by their size, distance from closest apex and center height (Fig. 3m, p, q). This segregation in spiral location and morphology is evidence that Snf7 spirals prefer to assume out-of-plane deformed, radially symmetric topologies. When comparing the position of the center of mass (COM) of each spiral with the position of the apices, we find an even better match with the substrate topography (Fig. 3k, m, p, q, dashed blue lines), indicative that it is the supra-molecular spiral structure and not the subunits in the innermost ring that dictate topological sensitivity.

If the spirals were constituted of a highly flexible polymer, they could change the sign of the radius of curvature without incurring a large energy penalty. Conversely, if the mechanical properties of the spiral constrained it to a smaller number of possible low-energy states, separate spiral populations of distinct topography would be observed. Clearly, the latter is the case (Supplementary Fig. 3). The two distinct spiral architectures were both characterized by non-interchanging curvature, and by adopting their size to the theoretical projected areas of their surface topography, 18,200 nm^2^ (theoretical value: ~17,700 nm^2^) for apical, and 3600 nm^2^ (theoretical value: ~3030 nm^2^) for interstitial spirals, respectively (Fig. 3m). In summary, the Snf7 spiral assembly morphology strongly depended on the prominence of the membrane topography.

### Membrane curvature-driven spiral reshaping

**Fig. 4:**
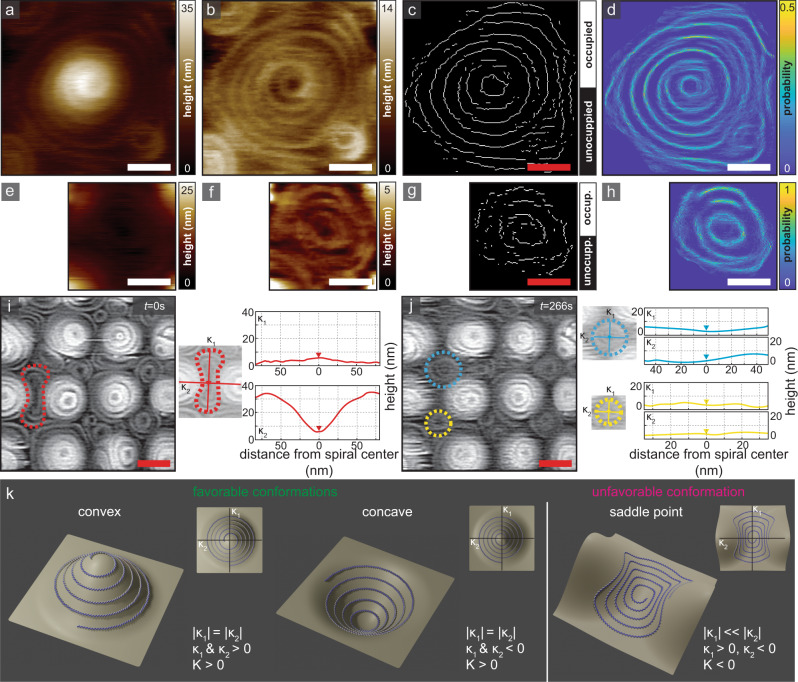
Membrane curvature-dependent Snf7 spiral stability and reshaping. **a** HS-AFM, **b** Gaussian fitting flattened, **c** binarized, and **d** probability map of a single, large apical Snf7 spiral. The spiral displayed no reshaping over ∼5 min HS-AFM observation (Supplementary Movie 4). **e** HS-AFM, **f** Gaussian fitting flattened, **g** binarized, and **h** probability map of a single interstitial Snf7 spiral. The spiral displayed no reshaping over the ∼2 min HS-AFM observation (Supplementary Movie 5). Scale bars: (**a**–**h**) 50 nm. **i**, **j** HS-AFM images of Snf7 spirals on an undulated support. A spiral on a saddle-shaped region (outlined in red in **i**) remodels into interstitial spirals (yellow and cyan in **j**) (Supplementary Movie 6). The line profiles (right) show the principal curvatures of the spirals. **k** Schematic illustrating an isolated Snf7 spiral adapting to convex and concave membrane geometry with rotationally symmetric principal curvatures (left and center, respectively) and a saddle point, where principal curvatures are of opposite signs and different magnitude (right). Source data are provided as a Source Data file.

To gain insights how spiral architecture and stability are influenced by the support topography, we acquired high-resolution time-lapse data of Snf7 spirals (Fig. 4, Supplementary Fig. 6). HS-AFM movie frames of an individual Snf7 spiral centered on a substrate apex (Fig. 4a) were fitted with a 2D-Gaussian, which was then subtracted from the raw data to remove the substrate topography and facilitate analysis of the protein assembly (Fig. 4b). The fine structural details in the HS-AFM topographs were extracted through skeletonizing and binarizing each frame (Fig. 4c). Summing and normalizing the binarized protein channel from individual frames resulted in a probability map (Fig. 4d), that showed little to no remodeling and high structural stability of apical spirals over the duration of 290 s (Fig. 4d, Supplementary Movie 4). The same is true for the interstitial spirals (Fig. 4e–h, Supplementary Movie 5). On the other hand, spirals that formed in saddle-shaped positions, with principal curvatures of opposite signs, were unstable and remodeled during imaging, ultimately forming smaller spirals nested within the concave interstitial areas (Fig. 4i, j, Supplementary Movie 6).

On non-planar surfaces two, apparently contradictory, observations were made: (i) Snf7 is membrane curvature insensitive at the molecular scale, in agreement with the absence of an amphipathic lipid-packing sensor (ALPS) domain in their sequence^36^, and (ii) Snf7 spirals adapt to the surface topography. Membrane curvature sensitivity is thus an emergent property of Snf7 filaments. However, there is no preference for concave versus convex shape. On the other hand, the inversion of convex to concave shape is prohibited, based on two observations: (i) spirals extend on undulated surfaces rather precisely to the size until the substrate would impose curvature inversion. In this work, this meant that convex spirals were significantly larger than concave spirals; however, both population had rather homogenous size distributions. (ii) In time-lapse observations, spiral remodeling was observed only in spirals that grew on areas with curvature inversion. Thus, the instability of saddle-shaped spirals was in stark contrast to the stability of radially symmetric apical and interstitial spirals, likely due to the fact that the same filament was forced to adapt to changing signs of principal curvatures (Fig. 4k, Supplementary Fig. 1). Taking these observations together, we propose that the Snf7 filament has high torsional rigidity and that this is a major driving factor for membrane curvature sensing and adaptation^42^.

### Snf7 filaments minimize energy in non-crowded conditions

So far, we have shown that Snf7 spirals sense and adapt to non-planar geometry, minimizing stress in the filament. In contrast, Snf7 spirals constrained to planar geometry suffer stress as a result of the deviation from a preferred conical or helical architecture with intrinsic helical pitch and/or tilt angle with respect to the surface^30,42,43,47^. Previous studies have given estimates for the preferred radius of curvature of Snf7 filaments^18^, including a study from our group, wherein the HS-AFM tip was used as a nanoscalpel in order to dissect Snf7 filaments and observe spiral remodeling^30^. However, the radius of curvature of spirals growing and remodeling in crowded conditions could be influenced by steric hindrance from neighboring spirals. Therefore, we decided to observe Snf7 spirals on flat SLBs at sub-saturating conditions.

**Fig. 5:**
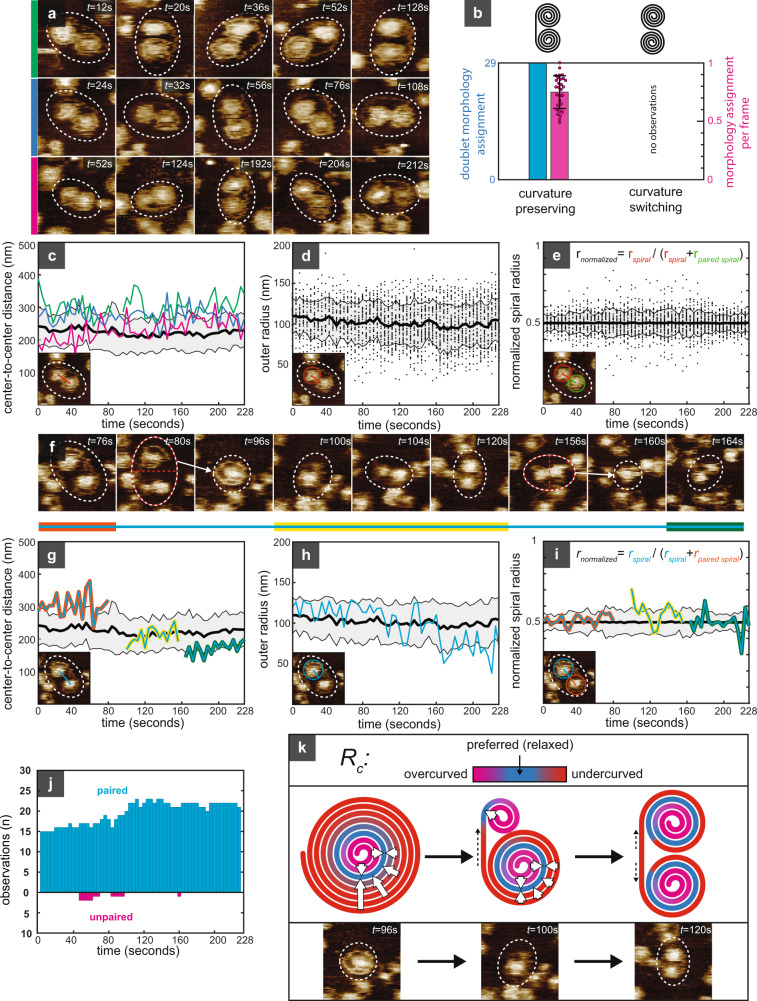
Snf7 filaments minimize bending energy in non-crowded conditions. **a** Time-lapse HS-AFM images of three representative spiral doublets (Supplementary Movie 7). **b** Morphology assignment of the doublets; curvature-preserving morphology could be assigned to all doublets at least once (cyan), and to 75 ± 14% of assemblies in each frame (magenta). No S-shaped doublets were observed. Error bars represent mean ± standard deviation of per-frame morphology assignment for *n* = 29 spiral doublets; magenta dots indicate percentage of frames in which morphology could be clearly assigned for each individual assembly. **c** Center-to-center distance between paired spirals. Colored lines show traces of doublets in **a** (mean ± sd of all observations: 225 ± 7 nm). **d** Distribution of spiral outer radii (black dots) over time. **e** Symmetry of spiral doublets: Distribution of normalized spiral radii (black dots) over time. **f** Time-lapse of a spiral doublet (dotted outline) that splits into one new spiral doublet and an isolated spiral (*t* = 96 s), which eventually reestablished doublet symmetry (*t* = 120 s), splits again into two doublets, which relaxed into symmetrical doublets (*t* = 164 s). Cyan line: spiral followed throughout the experiment. Multi-colored lines (cyan with red, yellow and green): periods reestablishing spiral pairing. **g** Multi-colored lines: center-to-center distance of successive doublets in **f** over time. Background trace as in **c**. **h** Outer radius of spiral in **f** over time. Background trace as in **d**. **i** Normalized spiral radius of the doublets formed by the spiral in **f**. Nascent spirals grow at the expense of the pre-existing spirals, reestablishing symmetry. **j** Histogram over time of paired (cyan) and unpaired (magenta) spirals. Unpaired spirals are only short-lived. **k** Middle: schematic of the proposed mechanism of doublet formation: Deviation from preferred spiral radius is penalized (red) and unpaired spirals rearrange into doublets to minimize undercurved filament length, thus reducing stress. Top: false-color scale for representation of local radius of curvature. Bottom: Experimental example of filament minimizing bending penalty. **c**–**e**, **g**–**i** Thick black lines and shaded areas represent mean ± sd of doublets in each frame. Dotted outlines of doublets or individual spirals are a guide to the eye. Source data are provided as a Source Data file.

After SLB formation on mica, we titrated up to ~300 nM Snf7 into the imaging solution of our HS-AFM (2-fold less concentrated than in our former HS-AFM work^30^). After a lag period, individual Snf7 assemblies diffusing across the SLB were observed. Strikingly, the shape of the assemblies was drastically different from that observed in crowded conditions: Snf7 assemblies formed stable spiral doublets (Fig. 5a, Supplementary Movie 7). There are two ways to form a spiral doublet: First, doublets are formed by a filament that curves only in one direction, in which case the sign of curvature over the entirety of its length is constant (curvature-preserving morphology), resulting in a C-shaped doublet. Second, doublets could be S-shaped. For S-shaped doublet two architectural models could apply, (i) a filament that changes the sign of in-plane curvature with a curvature inflection point between the two sub-spirals (bending-switching), or (ii) a filament that preserves the sign of in-plane curvature but features a torsional inflection point between the two sub-spirals (torsion-switching). Throughout 228 s of HS-AFM imaging (Supplementary Movie 7), we could assign the morphology of each doublet at least once (*n* = 24 doublet), and the orientation of the linker was discernible in 75 ± 14% doublets in each frame (*n* = 1123 observations). All doublets observed adopted the curvature-preserving morphology (Fig. 5b). From these observations, we concluded that the filament cannot switch curvature and does not allow torsional inversion. Instead, double-ended filaments curled into spiral doublets with preserved handedness (and, in extension, with one constant membrane-interacting face).

We obtained the size of each spiral in the doublets by binarizing the HS-AFM movies and reducing the spirals to binary disks (Supplementary Fig. 6). All the spirals in the doublets showed a narrow size distribution, and the separation distance between the paired spirals remained rather constant, averaging at ~225 nm, over the experiment (Fig. 5c). In agreement, the outer radii of individual spirals showed little variation, averaging at ~105 nm (Fig. 5d). Intriguingly, all doublets showed a high degree of symmetry, with both spirals in each doublet having approximately the same size (Fig. 5e).

Occasionally, doublets split, always through breaks in the filament linking the spirals together (Fig. 5f). Strikingly, none of the individual spirals thus formed remained stable; instead, the resulting individual spirals dynamically remodeled into new, smaller doublets. Accordingly, the center-to-center distance within the new doublet decreased (Fig. 5g), and so did the outer radius of the initial spiral (Fig. 5h). Concomitantly, the radius of the nascent spiral in the newly formed doublet increased, and the normalized radii of spirals within the doublets invariably reverted to ~0.5 as symmetry was reestablished (Fig. 5i). Thus, even when a free filament end is exposed at the periphery of an existing spiral and could grow through addition of Snf7 monomers, the preferred alternative is to remodel the spiral into a new, smaller doublet, where the nascent spiral feeds filament off of the preexisting spiral, and no stable individual spirals were observed (Fig. 5j).

We interpret these observations as a direct manifestation that the filaments have a preferred radius of curvature *R*_(preferred)_ (Fig. 5k, top). If a spiral exceeds *R*_(preferred)_, the outer turns will be undercurved and compress the inner turns to be overcurved. Upon further growth of the spiral, many more undercurved, higher-energy state, filament stretches are added (Fig. 5k, left). In a more natural setting, a lower-energy state could be achieved by out-of-plane motion and widening of the inner, overcurved turns, and a concomitant narrowing of the outer turns. This is, however, prevented on the rigid substrate. To avoid stress accumulation (on flat rigid surface), the undercurved turns can nucleate a new sister-spiral with *R*_(preferred)_, an energetically highly favorable process (Fig. 5k, middle). This process proceeds until the two spirals are equilibrated (Fig. 5k, right). Thus, a filament composed of *n* monomers in which *m* monomers have to comply to *R* > *R*_(preferred)_ (energetically unfavorable), will redistribute its content into two sub-spirals of *n*/2 monomers and thus in total two times more monomers can adopt to *R*_(preferred)_ and two times less have to comply to *R* > *R*_(preferred)_, neglecting the interconnecting strand. We reason that doublet splitting is energetically favorable until the outer turn of each sub-spiral is close to *R*_(preferred)_, when it will stop, which is at *R* ~30 nm^30^. This value is in good agreement with previous estimates of ~27 nm for *R*_(preferred)_ for Snf7^25,30^ and ~35 nm *R*_(preferred)_ for the *C. elegans* Snf7 ortholog Vps32^18^.

Under conditions where Snf7 filaments were not crowded on the membrane, we observed that filaments redistributed their content into C-shaped spiral doublets. The two sub-spirals equilibrate to the same radius of curvature, direct evidence that a large radius of curvature is energetically costly. Furthermore, S-shaped doublet spirals were not observed. From these results, we concluded that Snf7 filaments had indeed a preferred radius of curvature much smaller than the typical size of spirals, and the unique handedness of the C-shaped doublet spirals on the membrane indicated that the filament had only one membrane-binding face and torsional stiffness. It is important to note, however, that the doublets formed here represent only an energy minimization on a flat rigid support. If a filament was allowed to go out of plane, it can potentially form a corkscrew in which more turns are close to *R*_(preferred)_, as observed by electron microscopy^48^. Thus, removing the constraint of the rigid substrate should allow a larger number of spiral turns to adopt to *R*_(preferred)_ by forming a cone. We therefore proceeded to test the ability of Snf7 spirals to deform the membrane on a non-rigid support.

### A soft sample support

**Fig. 6:**
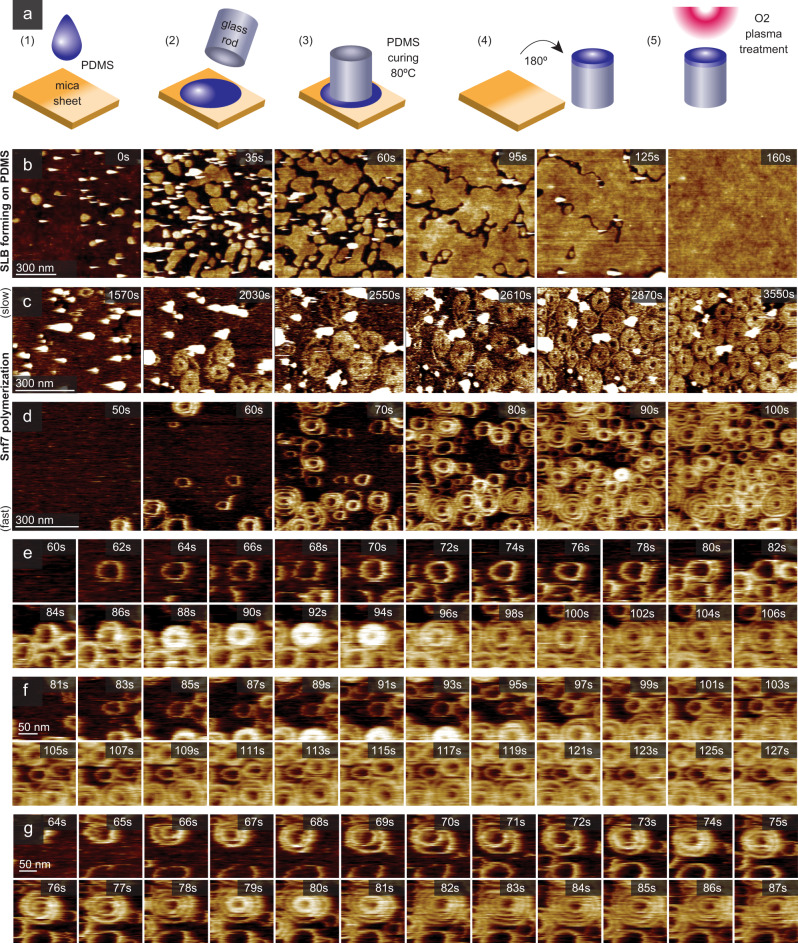
PDMS sample support design, SLB formation and Snf7 spirals on a soft surface. **a** Preparation of a soft PDMS sample support for HS-AFM. (1) A PDMS drop is pipetted onto atomically flat mica. (2) The HS-AFM sample stage is deposited on the PDMS droplet. (3) PDMS curing. (4) HS-AFM sample stage with PDMS layer is separated from mica sheet. (5) PDMS surface is O_2_-plasma treated to enhance hydrophilicity. The PDMS had a Young’s modulus of ~270 kPa (Supplementary Fig. 4). **b** SLB formation through SUV spreading and fusion (Supplementary Movie 8). **c** HS-AFM images of slow Snf7 polymerization on a PDMS-SLB (Supplementary Movie 9). **d** HS-AFM images of rapid Snf7 spiral polymerization on a PDMS-SLB (Supplementary Movie 10). HS-AFM time-lapse of a Snf7 spiral (**e**) polymerizing into a spiral of increased protrusion height, followed by a collapse into plane and spiral growth, (**f**) forming a closed stunted Snf7 ring unable of further growth, and (**g**) polymerizing into a large spiral displaying dynamics of the innermost turn.

Polydimethylsiloxane (PDMS) is a well-established method to uncouple SLBs from rigid support^49^, yet it has, to the best of our knowledge, not been used for biomolecular studies by AFM. To obtain a flat PDMS substrate layer, we used a freshly cleaved, atomically flat mica sheet as a mold. In brief (Fig. 6a; see Methods): a drop of fluid PDMS is pipetted onto a mica sheet (step 1), the HS-AFM sample stage glass rod is pushed top-down into the PDMS drop (step 2), followed by PDMS curing at 80 °C (step 3). After curing, the HS-AFM sample stage is removed from the mica and turned over to expose the flat PDMS (step 4), which is then placed in a vacuum chamber for oxygen plasma treatment (step 5). As a result, we obtain a ~10 µm thick hydrophilic PDMS film as sample support. Oxygen plasma treatment was found essential for lipid bilayer spreading, as previously reported^49,50^. The elastic properties of the PDMS support were determined using AFM elasticity measurements (Supplementary Fig. 4). Shortly after plasma treatment, the sample stage was mounted in the HS-AFM and a SLB formed on the PDMS through SUV (DOPC:DOPS, 6:4) fusion - a process that was directly monitored by HS-AFM (Fig. 6b, Supplementary Movie 8). Immediately following the SLB formation, Snf7 was added to the fluid cell at a final concentration of ~3 µM. Occasionally, the Snf7 polymerization process proceeded slowly and displayed the doublet stage (as observed on a mica-SLB, Fig. 5) before crowding was achieved ~50 min after Snf7 addition, at which point the connection between the individual paired spirals is no longer discernible (Fig. 6c, Supplementary Movie 9). However, in the overwhelming majority of cases, after an initial lag period, Snf7 spirals formed rapidly on the PDMS-supported lipid bilayer (PDMS-SLB), and within a minute the entire imaging area was covered by Snf7 spirals (Fig. 6d, Supplementary Movie 10). Snf7 spiral polymerization usually started with individually discernible single filament rings that formed spirals through continued polymerization (Supplementary Movie 10). However, certain Snf7 spirals seemed to stack vertically before collapsing and growing laterally (Fig. 6e). We also observed stunted Snf7 rings that did not grow further, remaining annular throughout the experiment (Fig. 6f). Finally, we observed Snf7 spirals with protruding inner rings that collapsed towards the substrate plane (Fig. 6g).

### Snf7 spiral loading on a soft membrane

**Fig. 7:**
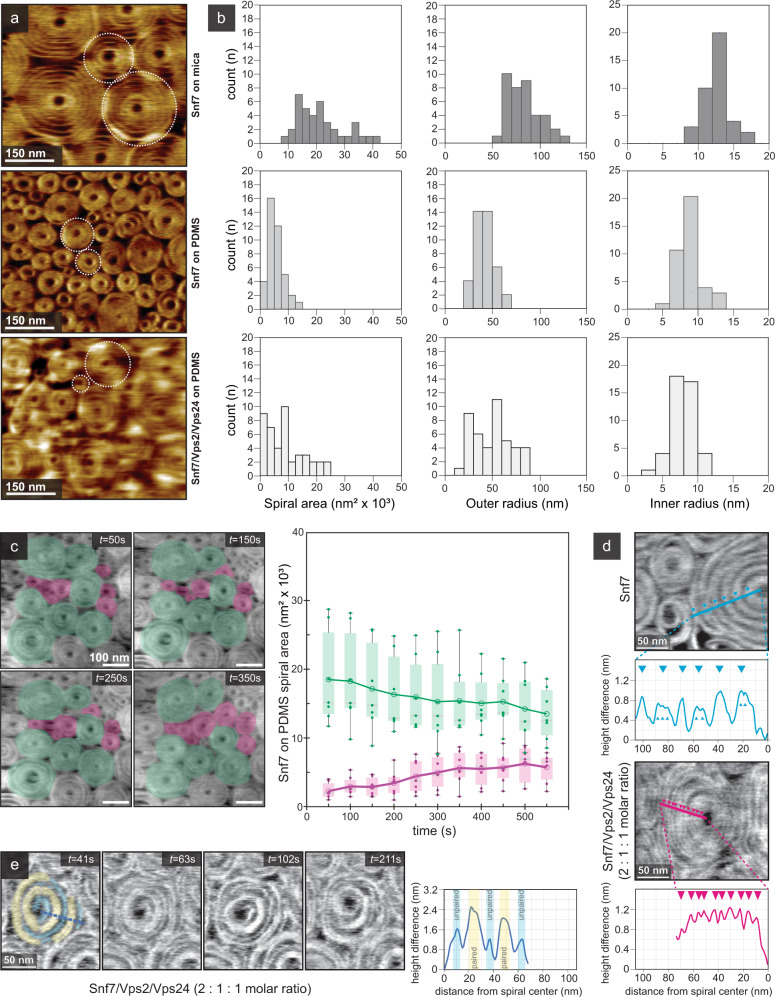
Snf7 spiral dynamics on SLBs on a soft substrate. **a** HS-AFM image of Snf7 spirals on mica (top), on PDMS (middle) and on PDMS after addition of Vps2/Vps24 to pre-polymerized Snf7 spirals (bottom). **b** Area (left), outer radius (center) and inner radius (right) distributions of Snf7 spirals on mica (top), on PDMS (middle) and on PDMS after addition of Vps2/Vps24 to pre-polymerized Snf7 spirals (bottom). **c** Left: HS-AFM time-lapse images of Snf7 spirals on PDMS-SLB (top right corner: time after Snf7-addition; overlays: spirals formed during initial polymerization (green, *n* = 7) and newly formed spirals (magenta, *n* = 7)(Supplementary Movie 11). Right: Box plot of Snf7 spiral area over time. Individual spiral areas are plotted as dots. Shaded boxes indicate 2^nd^ and 3^rd^ quartile. The whiskers of each boxplot extend to the most extreme data points. Empty circles connected by lines are the mean of each group; median of each group is represented by faint gray lines. **d** Top: Snf7 spiral on PDMS-SLB with line profile (cyan) showing height of spiral turns. Large cyan triangles show centers of turns, small cyan triangles point towards minor peaks within turns, corresponding to individual strands. Bottom: Spiral on PDMS-SLB after Vps2/Vps24 addition to pre-polymerized Snf7 spirals with line profile (magenta) showing height of individual spiral turns. Magenta triangles point towards individual filament peaks. **e** Left: Time lapse of individual spiral after addition of Vps2/Vps24 to pre-polymerized Snf7 spiral. Yellow overlays denote turns consisting of paired filaments, light blue overlays denote turns consisting of unpaired filaments. Right: height profile of the spiral taken at *t* = 41 s, with higher peaks (yellow) corresponding to paired filament turns, and lower peaks (light blue) corresponding to unpaired filament turns. Source data are provided as a Source Data file.

Matured Snf7 spirals formed on SLBs on soft PDMS revealed visually perceivable differences as compared to spirals grown on SLBs of the same composition on mica (Fig. 7a). On mica, commonly used in AFM studies, the Snf7 spirals had a wide size distribution averaging at ~20,000 nm^2^ (Fig. 7b, top left), and the outer and inner radii of the mature Snf7 spirals were 77 ± 26 nm (Fig. 7b, top center) and 11.4 ± 1.3 nm (Fig. 7b, top right), respectively. On PDMS, the average spiral area was only ~4000 nm^2^ (Fig. 7b, middle left), and the outer and inner Snf7 spiral radii were 42 ± 13 nm (Fig. 7b, middle center) and 8.3 ± 2.6 nm (Fig. 7b, middle right), respectively. Thus, on a soft support, matured spirals were smaller, and, in contrast to mica, the spiral turns were unresolved, suggesting a more compact filament packing. Disk-like, densely packed spirals were formerly observed on SLBs on mica when Snf7 was supplemented with other ESCRT-III components such as Vps2 and Vps24^24^, though these observations could be mechanistically unrelated. Because Snf7 spiral packing and morphology was different on PDMS-SLB as compared to spirals on mica-SLB, we wondered whether utilization of a soft substrate would also affect spiral packing and morphology after addition of other ESCRT-III components. Following addition of Vps2 and Vps24 to pre-formed Snf7 spirals on PDMS-SLB (Snf7:Vps2:Vps24 3 µM:1.5 µM:1.5 µM), we observed similarly dense spirals, with the spiral areas slightly increased compared to Snf7-only spirals on PDMS-SLB, though the spiral size distribution was still narrower and shifted towards smaller values as compared to Snf7 spirals on mica (Fig. 7b, bottom left). The outer radii of the spirals in presence of all three components were comparable to those of Snf7 alone (Fig. 7b, bottom center), while the inner radii were slightly smaller, further suggesting a more compact arrangement of the Snf7/Vps2/Vps24 spirals (Fig. 7b, bottom right, light gray).

HS-AFM time-lapse imaging showed that Snf7 spiral compaction on PDMS-SLB was a dynamic process: Snf7 spirals, initially resembling the assemblies observed on a rigid support, gradually decreased the inter-filament distance over time (Fig. 7c, Supplementary Movie 11). As existing Snf7 spirals on the PDMS-SLB decreased in size (Fig. 7c, green), new spirals formed in the area on the bilayer that was vacated by the densifying spirals (Fig. 7c, magenta). Coverage of the lipid bilayer by Snf7 thus remained roughly constant throughout the experiment, whereas the size of spirals converged towards the value shown in Fig. 7b (top left). No further change in spiral area was observed upon subsequent addition of Vps2 and Vps24 to already compacted Snf7 spirals. Occasionally, we obtained detailed information about the spiral architecture before and after Vps2/Vps24 addition to pre-formed Snf7 spirals (Fig. 7d). Snf7 on PDMS-SLB formed double-stranded filaments with inter-filament distance of ~14 nm (Fig. 7d, top), in agreement with previous estimates for Snf7 inter-filament distance^30^. Upon addition of Vps2/Vps24, the inter-filament distance was decreased to ~6.5 nm (Fig. 7d, bottom). Furthermore, filament pairs (Fig. 7e, yellow overlay) and single unpaired filaments (Fig. 7e, cyan) could co-exist and persist over time (Supplementary Movie 12).

The observation of dynamic Snf7 spiral densification (Fig. 7) was further evidence for the loaded spiral spring model^24,30,41,47^, in agreement with the equilibration of the spiral doublets (Fig. 6). According to this model outer filaments in Snf7 spirals relax towards a lower-energy state by decreasing their radius of curvature. However, compaction itself should be limited by volume exclusion between neighboring filaments in planar spirals;^30,37,51^ as filaments get closer, stacking and displacement in the vertical direction is expected. Thus, we wondered if Snf7 spiral compaction was sufficient and necessary for buckling against a soft membrane.

### Buckling of Snf7 spirals

**Fig. 8:**
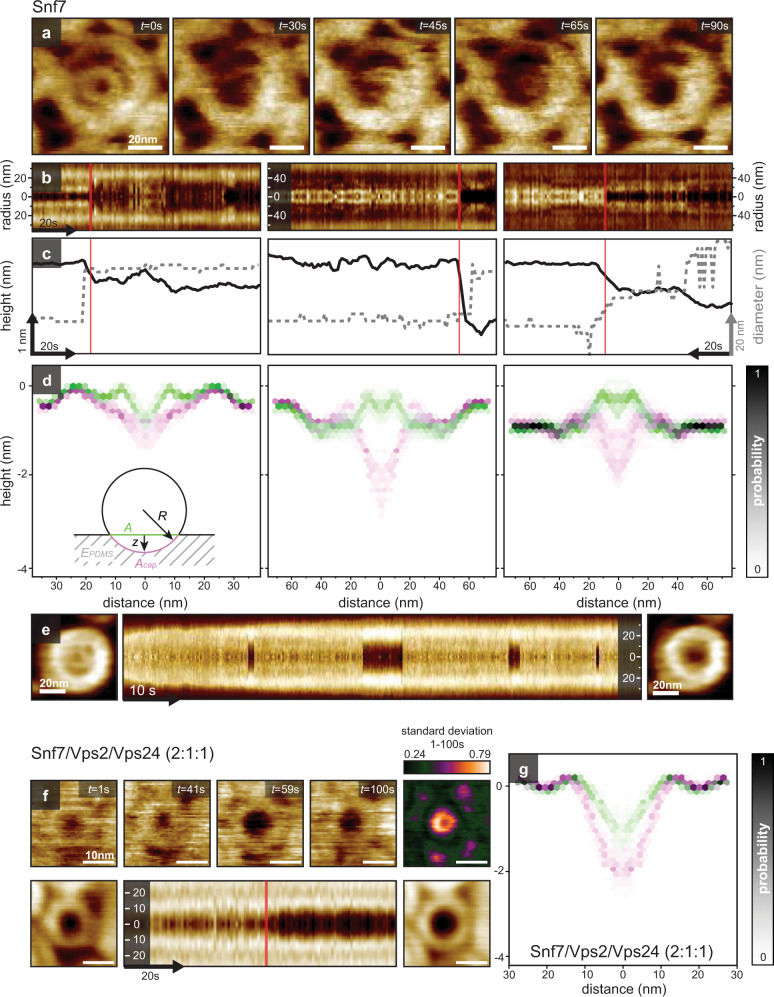
HS-AFM time-lapse images of a flat spiral to dome transition. **a** HS-AFM images of a Snf7 spiral. **b** Three kymographs across the spiral center extracted from three different spirals transitioning to a dome (Supplementary Movies 13–15). **c** Height profiles along the center in the kymograph (black). Diameter of the innermost Snf7 filament (gray dashed line). **d** Height profiles extracted from **b** before (green) and after (magenta) the transition. Inset: Spherical dome model for membrane deformation (same color code as the height profiles). **e** Snf7 spiral undergoing reversible out-of-plane into-plane transitions during HS-AFM movie acquisition (Supplementary Movie 16). Left: in-plane configuration. Right: out-of-plane configuration of the same spiral. Center: Kymograph of the spiral morphology over time. **f** Top: HS-AFM images of a pre-polymerized Snf7 spiral after addition of Vps2/Vps24 (Supplementary Movie 17). Top right: Standard deviation map of the HS-AFM images. Bottom left: in-plane configuration (average over 50 frames). Bottom right: Out-of-plane configuration (average over 50 frames). Bottom center: Kymograph of the spiral morphology over time. **g** Height profiles extracted from **f** before (green) and after (magenta) the transition. Source data are provided as a Source Data file.

Taking advantage of the unique capability of HS-AFM to acquire topographs at sub-second temporal and molecular lateral resolution, we were able to observe real-time Snf7 spiral buckling transitions on soft supports (Supplementary Movies 13–15). Typical events were characterized by the sudden disappearance of the innermost ring transitioning into an indentation (Fig. 8a), a phenomenon that is well represented in cross-sectional kymographs (Fig. 8b). As a result, the region where the innermost ring was located displayed a decrease in height while the apparent diameter increased (Fig. 8c). Probability density maps of the cross-section topography highlight the out-of-plane transition in these spirals (Fig. 8d). The innermost spiral ring could reversibly buckle out of plane and back into planar configuration as a function of time, corroborating that the observations of spiral buckling transitions indeed involved a rearrangement of filament and not a degradation process (Fig. 8e, Supplementary Movie 16).

Occasionally, structural details of the transition could be resolved (Fig. 8a): after out-of-plane motion of the innermost ring, a connecting stretch of the spiral filament was resolved visibly in a corkscrew configuration reaching from the spiral’s original membrane-supported plane downwards into the membrane and the soft support (Fig. 8a*, t* = 45 s). The center of the spiral showed continuous height decrease over the acquisition period (Fig. 8a, *t* = 90 s), suggesting that the post-transition configuration was a stable low-energy state. These observations represent, to the best of our knowledge, the first real-time molecular-level observations of a Snf7 spiral transition from a planar configuration to an out-of-plane buckled conformation. Given that Snf7 is the only protein component in these experiments, the observation that elastic energy amassed in the spirals through polymerization and released through buckling, provided direct evidence for the ‘loaded spring model’^18,30,41^, and showed that Snf7 alone was sufficient to initiate membrane deformation.

To test whether addition of Vps2/Vps24 to pre-polymerized Snf7 spirals affected the magnitude of the deformations we observed with Snf7 spirals on a PDMS-SLB, we supplemented the imaging solution containing Snf7 with Vps2/Vps24 at a 2:1:1 stoichiometric ratio. We observed events following a similar pattern as with Snf7-only spirals: the inner turn of spirals underwent an out-of-plane buckling transition, while the subsequent turn did not change height; this is perceived as a widening of the inner radius of the spiral after the buckling event, and is made evident by analyzing the standard deviation of the HS-AFM movies (Fig. 8f, Supplementary Movie 17). The morphology and extent of the observed buckling events were indistinguishable from the events observed for Snf7- only spirals (Fig. 8g).

We proceeded to estimate the necessary energy that the Snf7 spiral had to provide in the buckling transition, as:

1 $${G}_{{buckling}}={G}_{{membrane}-{bending}}+{G}_{{membrane}-{stretching}}+{G}_{{PDMS}-{compression}}$$

The first component characterizes the membrane deformation from flat (${R}_{0}=\infty$) to a spherical cap of radius $R$, and can be estimated following:

2 $${G}_{{membrane}-{bending}}={\kappa }_{{membrane}}/2\cdot A\cdot {\left(2/R-2/{R}_{0}\right)}^{2}=2A{\kappa }_{{membrane}}/{R}^{2}$$

where ${\kappa }_{{membrane}}$ is the membrane bending modulus of $\sim \!\!15{k}_{B}T$ as determined experimentally^52^, the area $A \sim 1520{{nm}}^{2}$ (*i.e*. area delineated by the innermost spiral turn radius $r$ after collapse), and the spherical cap radius $R$ is estimated from the innermost turn radius $r( \sim\!\! 22{nm})$ and the indentation depth $z( \sim\!\! 2{nm})$, $R={r}^{2}+{z}^{2}/2z=\, \sim\!\! 120{nm}$. Thus, ${G}_{{membrane}-{bending}} \sim 3{k}_{B}T$.

The second component addresses the energy cost to stretch the membrane of area $A \sim\!\! 1520{{nm}}^{2}$ into a spherical cap of area ${A}_{{cap}}=\pi \left({r}^{2}+{z}^{2}\right)=\, \sim\!\! 1535{{nm}}^{2}$, estimated by:

3 $${G}_{{membrane}-{stretching}}={k}_{A}/2\cdot {({A}_{{cap}}-A)}^{2}/A$$

where ${k}_{A}$ is the membrane stretch modulus of $\sim\!\! 100{pN}/{nm}$, as determined experimentally^52,53^. Thus, ${G}_{{membrane}-{stretching}} \sim 1.8{k}_{B}T$.

The third energy component concerns the compression of the layer of PDMS underneath the lipid bilayer, expressed by:

4 $${G}_{{PDMS}-{compression}}={E}_{{PDMS}}\cdot A\cdot {\triangle z}^{2}/(1-{\upsilon }^{2})\cdot 2\cdot {h}_{{PDMS}}$$

where ${E}_{{PDMS}}$ is the Young’s modulus of the PDMS as determined by AFM-based indentation measurements (Supplementary Fig. 4), $\upsilon$ is Poisson’s coefficient, ${h}_{{PDMS}}$ is the PDMS thickness as measured by AFM after scratching a defect into the layer, and $\triangle z$ is the indentation depth caused by the buckling event. Because the PDMS layer is thick (~10 µm) and the indentation shallow (~2 nm), the energy term to compress the PDMS underneath the bilayer is negligible, ${G}_{{PDMS}-{compression}}=\, \sim\! 0.4{k}_{B}T$.

Taken together, Snf7 had to deliver an estimated ${G}_{{buckling}}=5.2{k}_{B}T$. Such buckling was never observed on mica. The estimated energy cost of the buckling process is in a reasonable range for protein-mediated processes, but beyond a fortuitous observation of a thermal fluctuation. From these results we concluded that homopolymeric Snf7 spirals, when given the possibility, can generate force to undergo out of plane buckling.

## Discussion

Here, we provide conclusive evidence that individual homopolymeric Snf7 assemblies can act as loaded spiral springs on the underlying membrane. Depending on the rigidity of the support, Snf7 spirals will either adapt their conformation to the surface topography (Figs. 3 and 4), redistribute their filament content (Fig. 5), or load (Fig. 7) to remodel the underlying membrane and substrate (Fig. 8) to adopt an energetically favorable buckled conformation.

A considerable advancement in our understanding of the molecular mechanism of ESCRT-III mediated membrane deformation was provided with the loaded spiral spring model, which proposed that mechanical energy is stored in flat spirals that ultimately drives out-of-plane deformations^30,54^. Similar in mechanism, but with opposite starting and end points, it has been proposed that a dome or cone of ESCRT-III could collapse into a flat spiral, thus driving into-plane membrane deformation, and leading to membrane scission^21,41^. These two models of ESCRT-III function agree that there are at least two distinct conformations of ESCRT-III assemblies, a 2D-spiral and a 3D-cone. However, there is a disagreement about which architecture is the lower energy state. The data presented here show that spirals tend to adopt convex and concave geometries and that flat spirals containing filaments with low bending curvature are in a high-energy state. This provides evidence that spiral deformation in any direction is energetically more favorable than planar geometry, through minimization of low bending curvature filament stretches. Thus, the 3D-geometry represents an energy minimum of the ESCRT-III assembly. This is contradictory to previous reports of large unilamellar vesicles being flattened after incubation with human CHMP4B, and cryo-electron tomography reconstructions of such vesicles that showed CHMP4B assembled into rather flat spirals^55^. However, the flattened liposomes could have been deformed during the blotting of the cryo-electron microscopy grid, as the thickness of the film might have been less than the diameter of the vesicles. This deformation could increase their membrane tension, opposing or reverting buckled spirals to flat state. Furthermore, the out-of-plane spiral deformation events observed in our HS-AFM movies unambiguously demonstrate 2D to 3D spiral transitions with sub-nanometer vertical resolution (Fig. 8a, Supplementary Movies 13–17).

It is less clear how our observations relate to reports of the architecture of ESCRT-III assemblies on corkscrew-like membrane pipes as observed by cryo-electron tomography for CHMP4B-ΔC/CHMP2B-ΔC^55^ and Snf7/Vps2/Vps24^56^, which were proposed to be an extended buckled state. We have no knowledge about the architecture of Snf7 assemblies on such membrane pipe structures^56^, but it is possible that the spontaneous, frequent transitions from flat to curved spiral state as observed here, were stabilized into the buckled state by addition of Vps2-Vps24 (CHMP2-CHMP3), which would grow into cork-screw pipes in solution. While we observe Snf7 spiral out-of-plane deformation using HS-AFM, the limitations of HS-AFM as a surface-scanning technique would prevent us from obtaining detailed information if such a complex membrane shape were to form by further indentation into the PDMS support, regardless of the composition and stoichiometry of the ESCRT-III components. Nevertheless, the events observed in our study should correspond to a proposed first step in an ESCRT-III mediated membrane deformation sequence. In such a model, Snf7 initiates membrane budding that is then driven further out-of-plane by polymerization of additional ESCRT-III components, potentially replacing Snf7 through Vps4 ATPase action. It is important to note that all of the membrane pipes observed in the previous studies were formed in absence of Vps4 and ATP, thus representing energy minima of these protein complexes bound to membranes in bulk^55,56^. Hence, these studies also support that ESCRT-III components can drive membrane deformation without the use of chemical energy, in good agreement with our results, as in our case membrane deformation is solely driven by spontaneous polymerization of Snf7 into assemblies on PDMS-supported lipid bilayers.

Altogether, our results show conclusively that Snf7 can form energetically loaded higher-order structures, and that Snf7 polymerization can initiate the deformation of the underlying bilayer in absence of either an energy-providing molecular machinery or other ESCRT-III components. Further investigation at the molecular level into the interaction between Snf7 and other components of the ESCRT-III system, especially its direct downstream interactants Vps2 and Vps24, should prove crucial in understanding how these small-scale deformations are reshaped into large-scale, geometrically complex membrane-protein assemblies.

## Methods

### Protein expression and purification

Budding yeast (*Saccharomyces cerevisiae*) Snf7 (Addgene plasmid #21492), Vps2 (Addgene plasmid #21494) and Vps24 (kind gift from James Hurley lab, UC Berkeley, USA) were expressed and purified as previously described^40,57^. Proteins were expressed at 20 °C overnight in Rosetta2 (induction 0.5 M IPTG), before lysis by sonication (lysis buffer: 20 mM HEPES pH7.4, 100 mM NaCl, 1% Triton, cOmplete) and HisTrap purification (elution buffer: 20 mM HEPES pH7.4, 100 mM NaCl, 100 mM Imidazole). MBP-His-tag was cleaved off using TEV followed by purification on a Superdex 200 26/60 column (buffer: 20 mM HEPES pH 8.0).

### Liposome preparation for all HS-AFM experiments

The lipid vesicles used to prepare the lipid bilayer were prepared by mixing 1,2-dioleoyl-sn-glycero-3-phosphocholine (DOPC) and 1,2-dioleoyl-sn-glycero-3-phospho-L-serine (DOPS) (Avanti Polar Lipids, Alabama, USA) in chloroform in a 6:4 ratio (mol:mol) to a final concentration of about 0.33 mg/mL. The lipid mixture was evaporated in a 2.5 mL amber glass vial under a stream of argon. Mechanical agitation was applied concurrently with evaporation to ensure efficient spreading of a thin lipid film over the inner surface of the vial. The vial was placed under vacuum and desiccated overnight to remove leftover traces of chloroform. 1 mL of 150 mM potassium chloride (KCl), 10 mM tris(hydroxymethyl)aminomethane (Tris), pH 7.4 buffer heated to 85 °C was then used to rehydrate the lipid film. The suspension obtained was then agitated by vortexing for 45 seconds, followed by a 15 s heating step at 85 °C in a water bath. This cycle was repeated five times. We then used a probe sonicator to break down mechanically any multilamellar vesicles in the turbid lipid suspension, as well as to reduce the size of the lipid vesicles. The final, clear lipid suspension was added directly to the imaging chamber of the sample-scanning High-Speed Atomic Force Microscope (Research Institute of Biomolecular Metrology (RIBM), Japan).

### Polydimethylsiloxane (PDMS) substrate preparation

PDMS curing agent and PDMS elastomer base (Sylgard 184, Sigma-Aldrich) were mixed at a 1:50 weight ratio. The substrates were prepared following the steps depicted in Fig. 6a. A drop (~5 µL) of PDMS was deposited on a freshly cleaved mica sheet to ensure the flatness of the substrate and a clean HS-AFM sample stage glass rod (height: 2 mm; diameter: 1.5 mm) was placed on top. Next, the PDMS was cured in an oven at 80 °C for 4 h. Once the PDMS was cured, the glass rod was peeled from the mica. The PDMS has higher adherence to the rough glass rod surface than to the atomically flat mica surface. The PDMS films display a thickness of a few (~10) micrometers. O_2_ plasma treatment was used to increase the number of hydroxyl groups on the PDMS surface to render the surface hydrophilic, which was found essential for subsequent lipid bilayer spreading, as previously reported^49,50^. A JPK-Bruker Nanowizard was used to assess the Young’s modulus of the surface (Supplementary Fig. 4). Force curves were acquired with a 2 µm SiO_2_ sphere attached at the end of the cantilever (Sqube, NanoAndMore, USA). After PDMS substrate preparation, 2ul of liposomes (~1 mg/ml) were deposited onto the PDMS substrate. After 20 min, the sample was rinsed with imaging buffer (10 mM Tris at pH 7.4, 150 mM KCl). Alternatively, to observe how the SLB spreads on PDMS, 10 uL of liposomes (~1 mg/ml) were added directly to the imaging chamber and SLB spreading was observed until a confluent bilayer was obtained; the imaging solution was subsequently exchanged to remove excess unburst liposomes. After assessment of the surface and the supported lipid bilayer homogeneity, Snf7 was added to the fluid chamber to a final concentration of 3 μM.

### Periodically curved substrate preparation

To image Snf7 polymerization on curved lipid bilayer patches, we obtained HS-AFM calibration grids with well-defined lateral periodicity of 150 nm peak-to-peak distance (Model 150-2D, Advanced Surface Microscopy, Indiana, USA). The chemical composition of the substrate was silicon dioxide with protrusions consisting of aluminum. The calibration grids were treated with oxygen plasma in a plasma etcher at 300 mTorr pressure for 1 min to increase surface hydrophilicity and facilitate lipid deposition. Repetitive experimental use and exposure to oxygen plasma for cleaning transformed the initially cylindrical pillars into periodic bumps that further eroded with extensive experimental repetition, allowing preparation of undulated substrates of varying protrusion height. The imaging solution used for the experiment is identical to the buffer used for lipid vesicle preparation unless specified otherwise. The volume of lipid vesicle suspension used in each experiment varied from ~1/10 of the total imaging solution to ~4/10 of the total imaging solution. Lipid coverage of the curved substrate was enhanced by lowering the set point of the HS-AFM, thus exerting increased force on the lipid vesicles adhering to the substrate compared to common imaging conditions. To remove potential unburst lipid vesicles from the fluid chamber, we exchanged 5x the total volume of the imaging solution. After assessment of the supported lipid bilayer on the curved substrate and image acquisition area positioning, Snf7 was added to the fluid chamber to a final concentration of ~3 µM. After about 60 s Snf7 filaments and small rings begin to form on the SLB. Surface coverage with Snf7 saturates once Snf7 assembles into spiral structures (usually in less than 2 min).

### HS-AFM data acquisition and analysis

Imaging was performed at room temperature on an SS-NEX (RIBM) using short cantilevers (USC-F1.2-k0.15, NanoWorld, Switzerland) with spring constant of ~0.15 N/m and a resonance frequency around 0.6 MHz. The HS-AFM data was acquired using the Eagle software package by RIBM in an Igor Pro 7 environment (WaveMetrics, Lake Oswego, OR, USA). Pixel sampling ranged between 250 × 250 pixels and 300 × 300 pixels, and frames were acquired at rates ranging from 0.2 to 1 frames per second. Movie alignment was carried out using either the StackReg plugin^58^ for Fiji or custom Matlab (MathWorks, Natick, Massachusetts) scripts written in-house for this specific purpose.

#### Thresholding algorithm

To detect the protein polymerization among the dominant surface features of the non-planar support, as in Fig. 2, we applied a custom adaptive thresholding algorithm (Supplementary Fig. 2). We approximated the value of the substrate surface at any position by a 3D-Gaussian weighted average of the local neighborhood of the pixel (kernel size 5 pixels × 5 pixels × 5 slices, sigma = 0.5). The surface obtained in this way for every single frame was then rescaled proportionally to the height of the original data, thereby obtaining the support channel (Fig. 2b, green). The Snf7-channel (Fig. 2b, c, magenta) was obtained by subtracting the support channel from the raw data, thus enhancing the discernibility of support and protein. The separation method to obtain the protein channel results in non-linearity of protein channel pixel intensities between the topographically different parts of the substrate, still preventing binarization utilizing a global threshold value. To this end, the non-linearity was accounted for by sorting the pixels based on their intensity in the support channel in ascending order, then applying the same sorting order to the Snf7 channel. A linear fit was applied to the sorted pixel intensities in the Snf7 channel and the result of the fit was set as threshold for binarization in the occupancy analysis of the protein channel. The intercept of the fit could be manually adjusted while keeping the slope of the threshold constant to optimize binarization of the protein channel until the structural features (i.e. turns) of Snf7 spirals could be resolved.

#### Surface parameters calculation

The background (substrate) channel was used to calculate surface parameters (height, slope, and curvature) from a reference frame before Snf7 polymerization, as in Fig. 2. Since the surface parameters are calculated from data that has not been affected by protein addition or filtering, the corresponding surface parameters represent more faithfully the actual topography of the surface than the background channel used for binarization. Pixel height values are the raw data height, slope values are calculated as the height gradient at each pixel, and curvature is calculated by treating the protrusions as spherical caps and determining their radius of curvature (Fig. 2h). Each row in the occupancy plots represents an individual pixel, sorted by either height, slope or curvature, and each column represents a timepoint in the experiment. The sorting order of the pixels is different for each surface parameter but does not affect the binary occupancy of the pixel by Snf7.

#### Spiral doublet analysis

HS-AFM images were binarized using a global threshold (Otsu method). Following, through pixel dilation and erosion in ImageJ the binarized spiral doublets were transformed into binary disks. Assuming each spiral (now binary disk) to be roughly circular, the outer radii of the spirals were calculated from the disk area (Fig. 5d, h). Spiral centers were manually selected for each frame and used to calculate the center-to-center distance of each spiral doublet (Fig. 5c, g).

### Reporting summary

Further information on research design is available in the Nature Research Reporting Summary linked to this article.

## Supplementary information

Supplementary Information

Description of Additional Supplementary Files

Supplementary Movie 1

Supplementary Movie 2

Supplementary Movie 3

Supplementary Movie 4

Supplementary Movie 5

Supplementary Movie 6

Supplementary Movie 7

Supplementary Movie 8

Supplementary Movie 9

Supplementary Movie 10

Supplementary Movie 11

Supplementary Movie 12

Supplementary Movie 13

Supplementary Movie 14

Supplementary Movie 15

Supplementary Movie 16

Supplementary Movie 17

Reporting Summary

## Data Availability

Any data relating to the findings presented in this Article are available within the article and its supplementary information files. Additional information and relevant raw data are available from the corresponding author upon reasonable request and at the earliest convenience. Source data are provided with this paper.

## Source data

Source Data

## Code availability

The code used to analyze the data as reported in Figs. 2 and 4 has been uploaded to GitHub and is available through the following link: github.com/Nebojsa-Jukic/Snf7_scripts
