# Supplementary material for: Snf7 spirals sense and alter membrane curvature
Source: Nat Commun. 2022 Apr 21;13:2174. doi: 10.1038/s41467-022-29850-z (PMC9023468; doi:10.1038/s41467-022-29850-z)
Supplement: Supplementary file 1 — Supplementary Information [file 41467_2022_29850_MOESM1_ESM.pdf]

# Snf7 spirals sense and alter membrane curvature

Nebojsa Jukic[1,#], Alma P. Perrino[2,#], Frédéric Humbert[3], Aurélien Roux[3,4] and Simon Scheuring[2,5,6]*

[1] Physiology, Biophysics and Systems Biology Graduate Program, Weill Cornell Medicine, New York City, NY 10065, USA
[2] Department of Anesthesiology, Weill Cornell Medicine, New York City, NY 10065, USA
[3] Department of Biochemistry, University of Geneva, CH-1211 Geneva, Switzerland
[4] Swiss National Centre for Competence in Research Programme Chemical Biology, CH-1211 Geneva, Switzerland
[5] Department of Physiology and Biophysics, Weill Cornell Medicine, New York City, NY 10065, USA
[6]Kavli Institute at Cornell for Nanoscale Science, Cornell University, Ithaca, New York 14853, USA
[#] These authors contributed equally
* Correspondence to: sis2019@med.cornell.edu

## Supplementary Information

## Supplementary Figure 1)

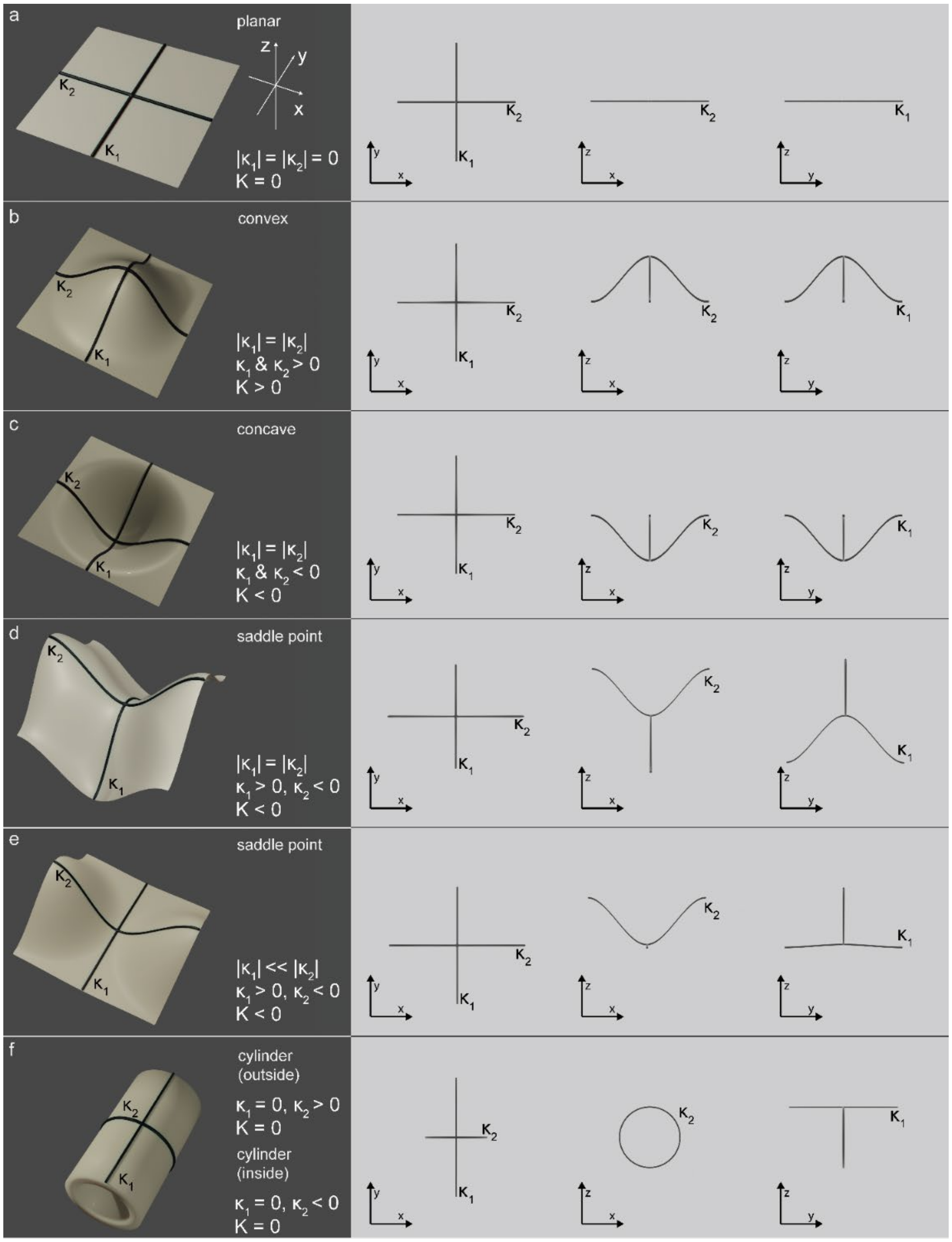


**Supplementary Figure 1: The different geometries of curved membranes used for observations of ESCRT assemblies on lipid bilayers. (a)** Planar supported lipid bilayers are often used in fluorescence microscopy and former HS-AFM studies of ESCRT proteins, but do not reflect the complexity of *in vivo* membrane geometry. The principal curvatures of the membrane ($\kappa_1$ and $\kappa_2$) are 0 and so is the Gaussian curvature ($K = \kappa_1 \times \kappa_2$). **(b)** Representation of a convex supported lipid bilayer, with both principal curvatures being greater than zero, resulting in a positive Gaussian curvature. **(c)** Representation of a concave supported lipid bilayer, with both principal curvatures being less than zero, resulting in positive Gaussian curvature. **(d)** A representation of a saddle point on the lipid bilayer, with principal curvatures of equal magnitudes but opposite signs, resulting in negative Gaussian curvature. **(e)** A representation of a saddle point on the lipid bilayer, with principal curvatures of opposite signs and different orders of magnitude, resulting in negative Gaussian curvature. **(f)** Representation of a membrane tube. One principal curvature of the tube is always equal to 0, and the other is greater than zero. This results in a Gaussian curvature equal to zero. In case where protein binds on the inside of the tube, the sign of the principal curvature along the perimeter of the tube is inverted, but the sign of principal curvature of the tube remains zero, thus preserving zero Gaussian curvature. The supports in this study were defined by a periodic arrangement of convexities and concavities; they can thus best be approximated as a mixture of the shapes described in **(b)**, **(c)** and **(e)**.

## Supplementary Figure 2)

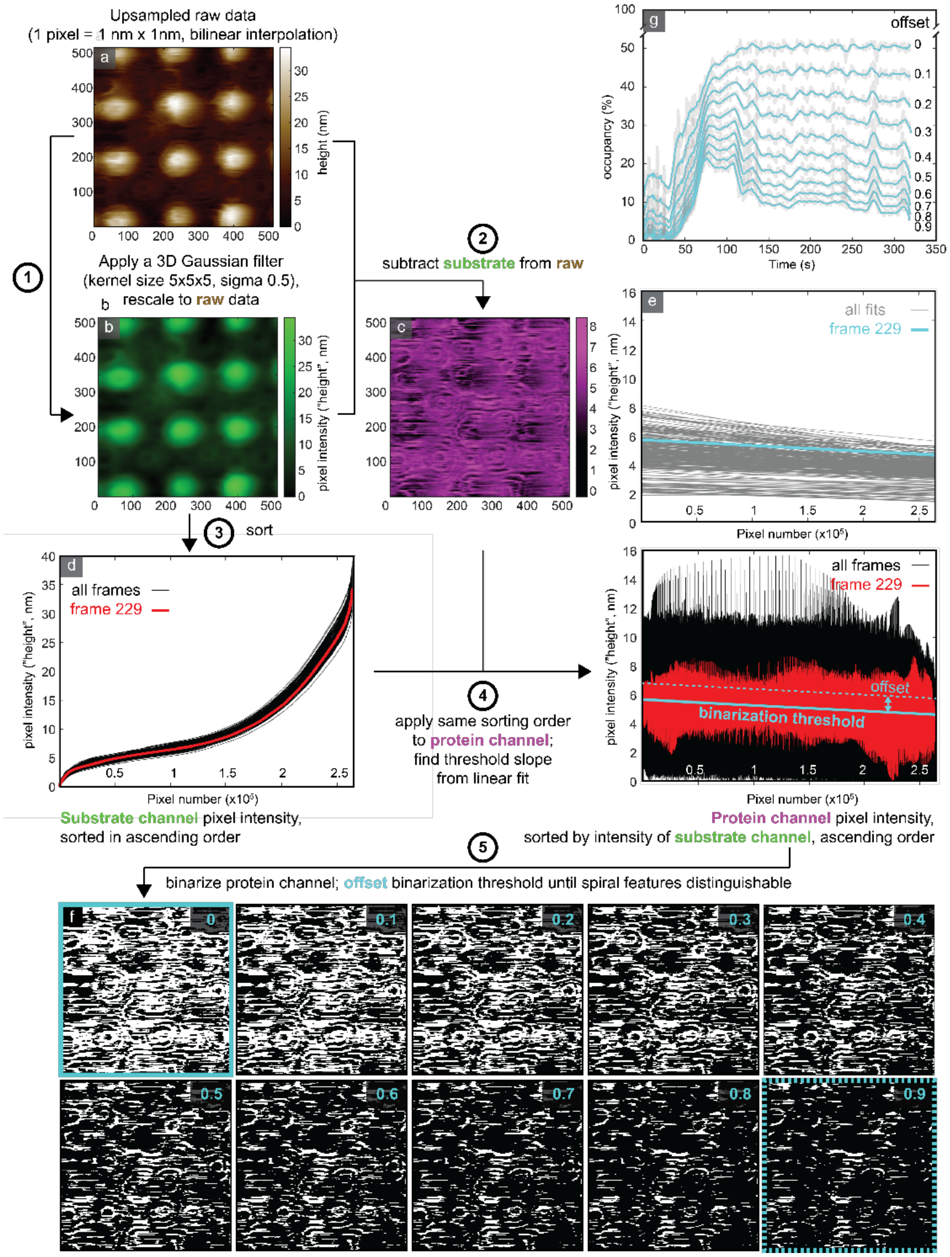


**Supplementary Figure 2: Adaptive thresholding algorithm and binarization for HS-AFM movies of Snf7 on SLB on nanopatterned support. (a)** HS-AFM raw data frames are first over-sampled to 1nm/pixel using bilinear interpolation. Then, a 3D Gaussian filter with a user-defined kernel size (5x5x5 kernel in this case) is applied, and the resulting image is rescaled to the height extrema of the original image to obtain the substrate channel (1). **(b)** Substrate channel of individual HS-AFM movie frame. The substrate channel is subtracted from the HS-AFM data to obtain the protein channel of the individual frame (2). **(c)** Protein channel of individual HS-AFM movie frame. **(d)** Intensity plot of the substrate channel of the HS-AFM movie, sorted by increasing intensity (3). The intensity of the substrate channel in frame 229 (shown in (b)) is plotted in red. **(e)** Applying the same sorting order as in (d) to the protein channel (c), we obtain the intensity profile for the protein channel (4). The residual non-flatness of the pixel intensities prevents application of global thresholding. By applying a linear fit to the intensity profiles of each individual frame (bottom), we obtain a sloped binarization threshold (cyan line). This procedure is applied to each frame (top), yielding a unique threshold for every frame. Step 5:Keeping the thresholding slope constant for each frame, the threshold can then be offset by an arbitrary value to fine-tune the level of detail that is preserved within spirals. **(f)** Examples of different offsets of the binarization threshold changes the ratio of occupied vs. unoccupied pixels. The optimal offset is picked based on how well spiral turns are resolved and separated. **(g)** Total occupancy profile shows the percentage of all occupied pixels as a function of time and thresholding offset. Source data are provided as a Source Data file.

## Supplementary Figure 3)

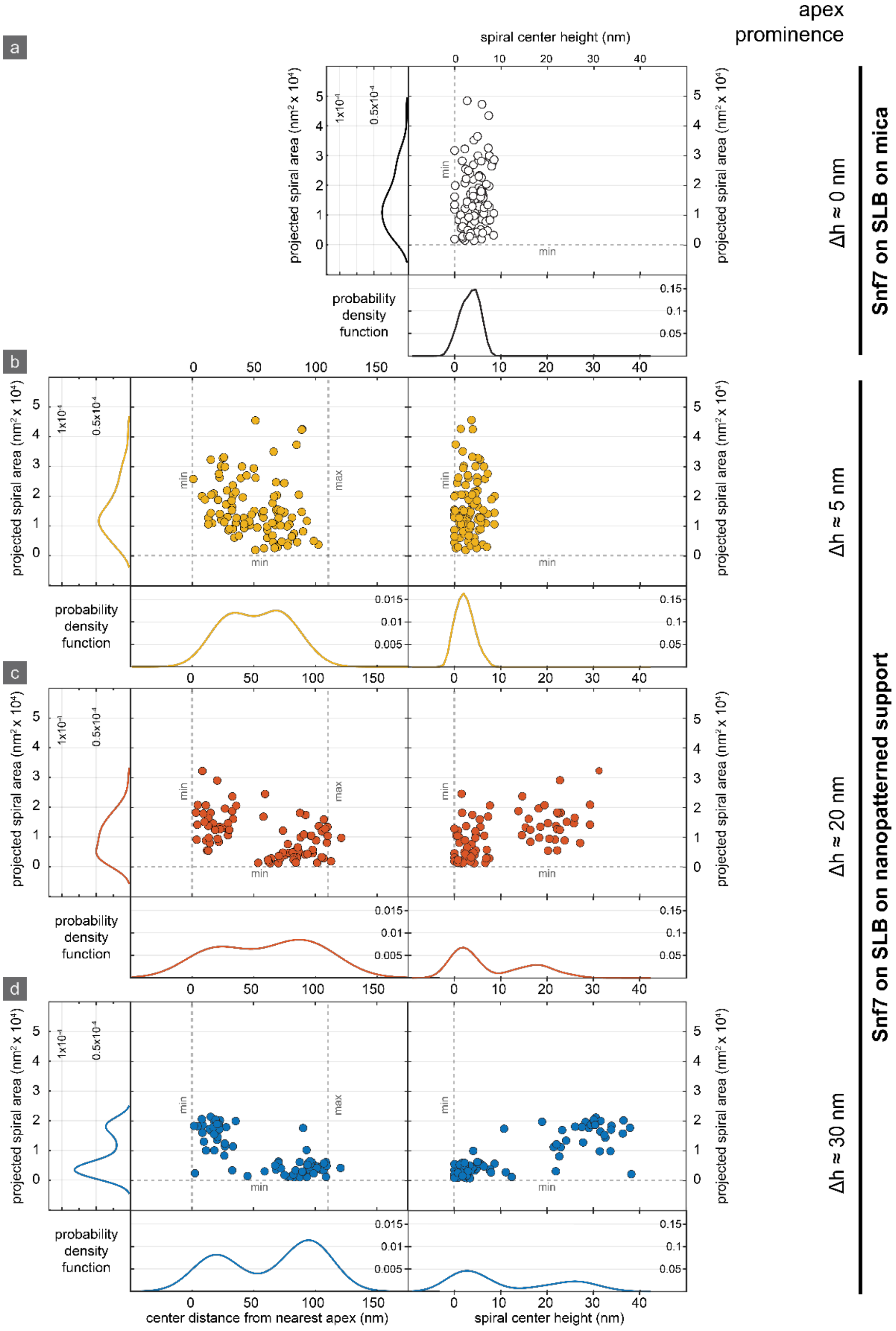


**Supplementary Figure 3: Snf7 spirals are membrane curvature sensitive (separation of plots shown in figure 3 in the main text). (a,b,c,d)** Distributions of 2D-projected Snf7 spiral areas on planar **(a)** and undulated supports of low (Δh ≈ 5 nm, **b**), moderate (Δh ≈ 25 nm, **c**), and prominent (Δh ≈ 35 nm, **d**) protrusions. Left: Scatter plots of Snf7 spirals, with 2D-projected area versus spiral center distance from nearest apex **(b,c,d)**. Right: Scatter plots of Snf7 spirals, with 2D-projected area versus spiral center height **(a,b,c,d)**. White: *n*=99 spirals, 5 different imaging areas on planar substrate (a). Yellow: *n*=98 spirals, 5 different imaging areas on substrate (b). Red: *n*=80 spirals, 4 different imaging areas on substrate (c). Blue: *n*=78 spirals, 5 different imaging areas on substrate (d). Source data are provided as a Source Data file.

## Supplementary Figure 4)

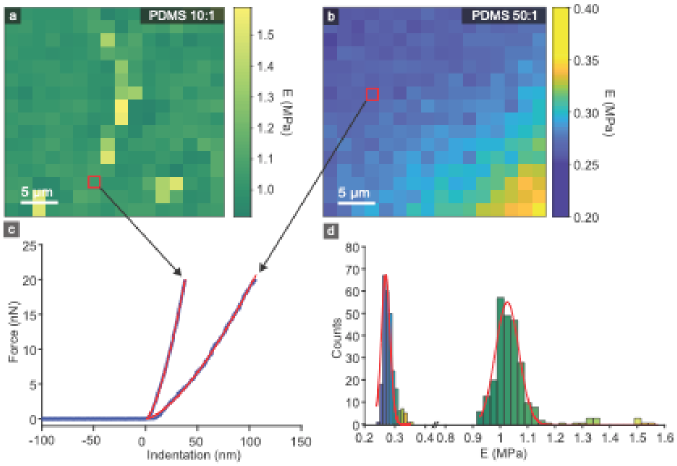


**Supplementary Figure 4: Determination of Young's modulus *E* of PDMS sample stages.** Force volume maps of **(a)** PDMS 10:1, and **(b)** PDMS 50:1 cured sample stages (the ratios 10:1 and 50:1 stand for the PDMS to crosslinker ratio in the mixture). **(c)** Representative force-distance curves with Hertz-model fits (red) for representative regions on the two substrates. **(d)** Distribution of Young's moduli E on the two substrates. The PDMS used in the experiments in the main text was prepared at a PDMS to crosslinker ratio of 50:1 and had an average elasticity of 270kPa. Source data are provided as a Source Data file.

**Supplementary Figure 5)**

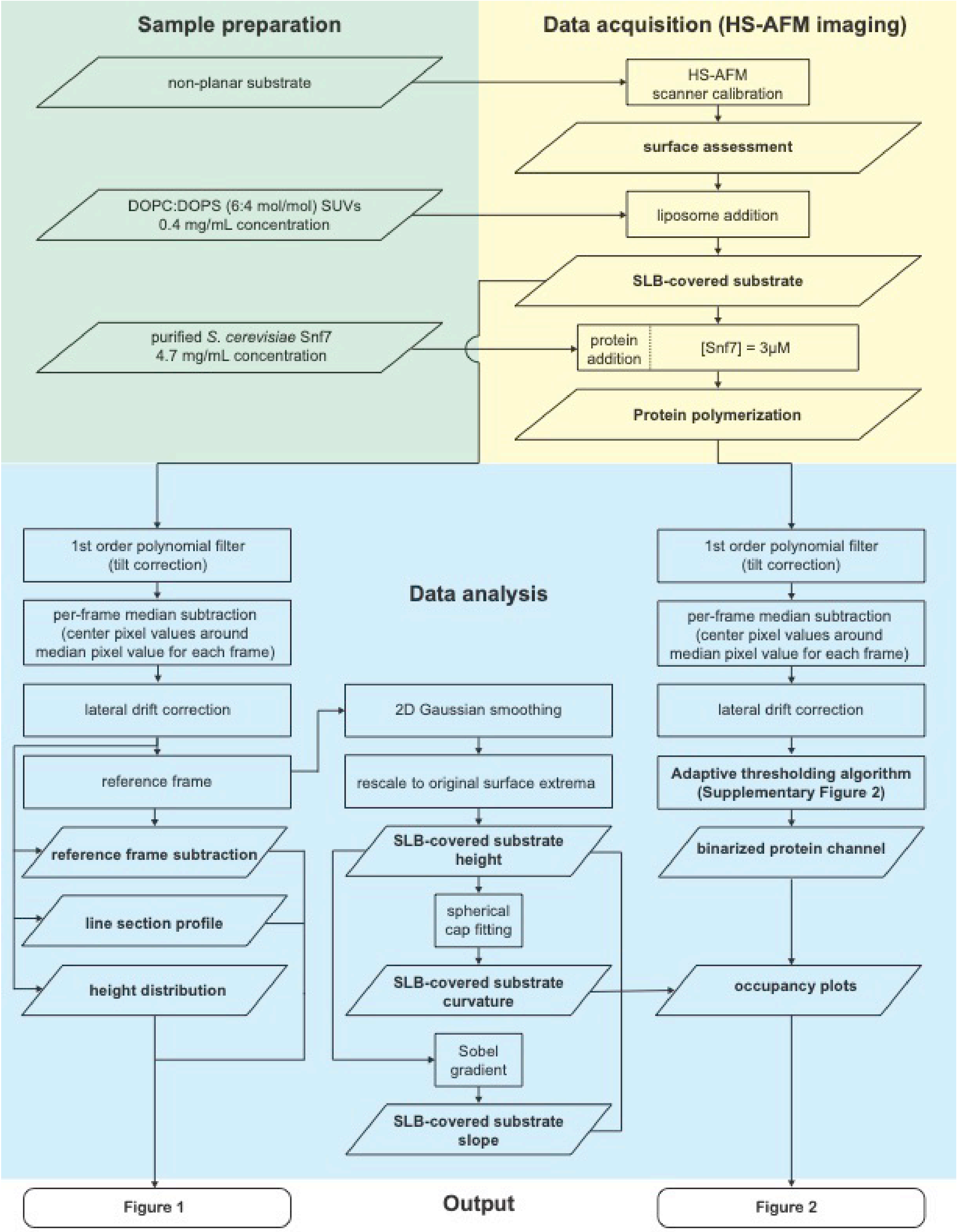


**Supplementary Figure 5: Analysis Figures 1 and 2.** Flowchart describing the individual steps in HS-AFM sample preparation (green), data acquisition (yellow), data analysis (cyan) for Figures 1 and 2.

**Supplementary Figure 6)**

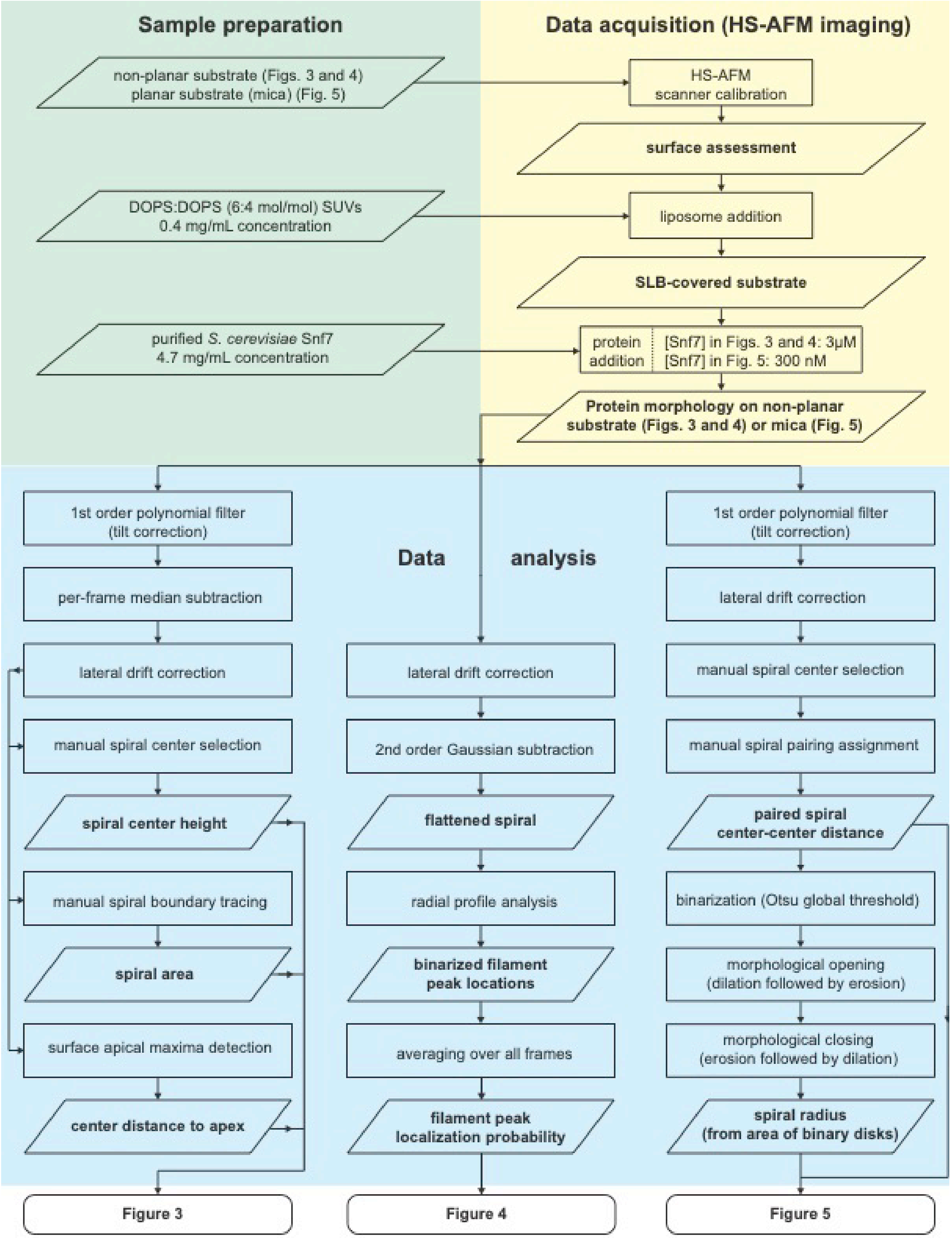


**Supplementary Figure 6: Analysis Figures 3, 4, and 5.** Flowchart describing the individual steps in HS-AFM sample preparation (green), data acquisition (yellow), data analysis (cyan) for Figures 3, 4, and 5.

**Supplementary Figure 7)**

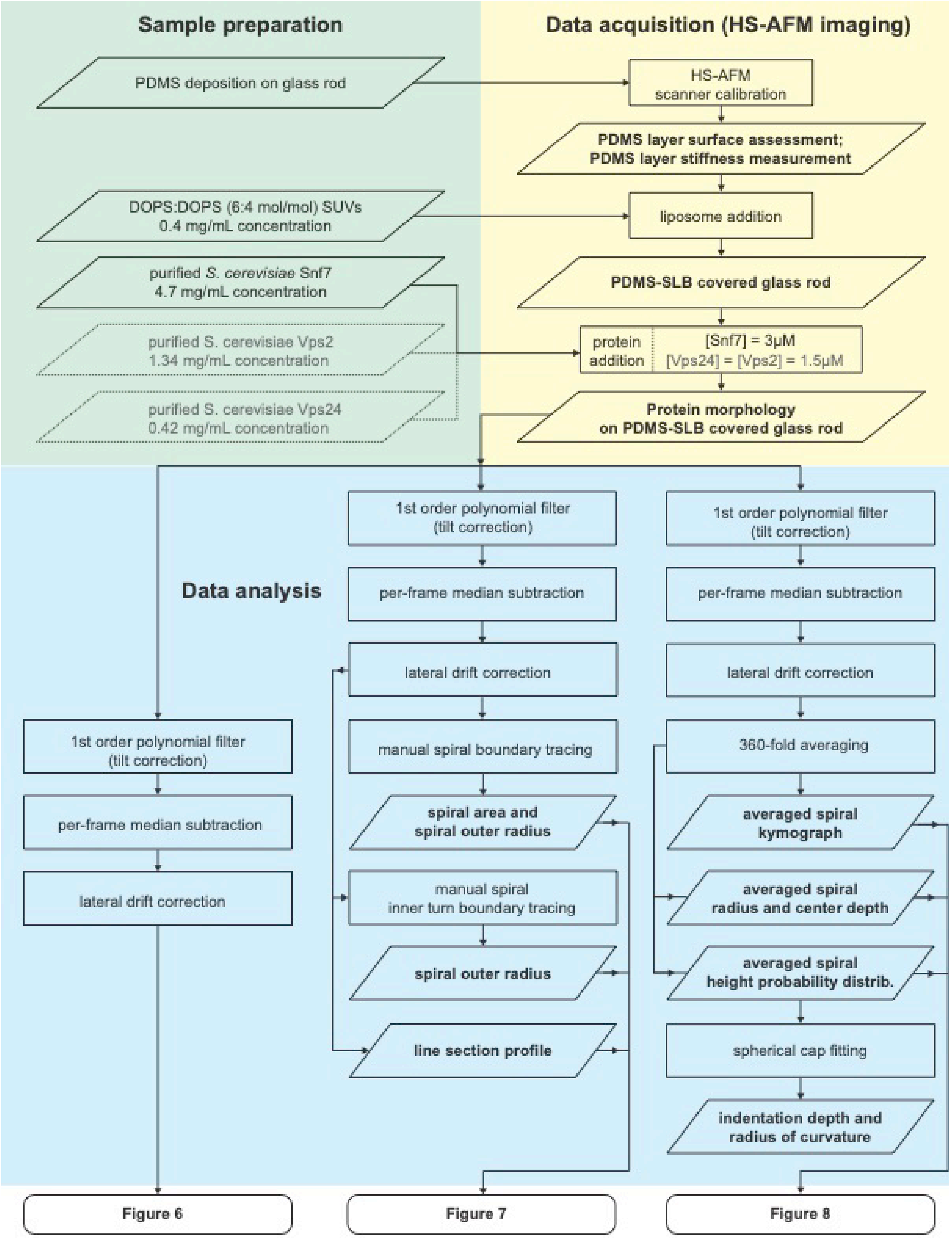


**Supplementary Figure 7: Analysis Figures 6, 7, and 8.** Flowchart describing the individual steps in HS-AFM sample preparation (green), data acquisition (yellow), data analysis (cyan) for Figures 6, 7, and 8.
