# Supplementary material for: Snf7 spirals sense and alter membrane curvature
Source: Nat Commun. 2022 Apr 21;13:2174. doi: 10.1038/s41467-022-29850-z (PMC9023468; doi:10.1038/s41467-022-29850-z)
Supplement: Supplementary file 2 — Description of Additional Supplementary Files [file 41467_2022_29850_MOESM2_ESM.pdf]

Description of Additional Supplementary Files

File name: Supplementary Movie 1

Description: High-Speed Atomic Force Microscopy movie of supported lipid bilayer spreading on a nanopatterned support

File name: Supplementary Movie 2

Description: High-Speed Atomic Force Microscopy movie of Snf7 polymerization on a supported lipid bilayer-covered nanopatterned support, with support channel (green) and Snf7 channel (magenta) separated for clarity.

File name: Supplementary Movie 3

Description: High-Speed Atomic Force Microscopy movie of Snf7 spiral on planar rigid supported lipid bilayer growing at the periphery.

File name: Supplementary Movie 4

Description: High-Speed Atomic Force Microscopy movie of apical Snf7 spiral on supported lipid bilayer-covered nanopatterned support, with substrate information subtracted for easier protein visualization.

File name: Supplementary Movie 5

Description: High-Speed Atomic Force Microscopy movie of interstitial Snf7 spiral on supported lipid bilayer-covered nanopatterned support, with substrate information subtracted for easier protein visualization.

File name: Supplementary Movie 6

Description: High-Speed Atomic Force Microscopy movie of substrate topography-dependent remodeling of a Snf7 spiral formed in a saddle point on a supported lipid bilayer-covered nanopatterned support.

File name: Supplementary Movie 7

Description: High-Speed Atomic Force Microscopy movie of Snf7 spiral doublets forming in non-crowded conditions on a mica-supported lipid bilayer.

File name: Supplementary Movie 8

Description: High-Speed Atomic Force Microscopy movie of supported lipid bilayer formation on a soft polydimethylsiloxane layer.

File name: Supplementary Movie 9

Description: High-Speed Atomic Force Microscopy movie of Snf7 spiral doublets forming during slow Snf7 polymerization on a polydimethylsiloxane-supported lipid bilayer (PDMS-SLB).

File name: Supplementary Movie 10

Description: High-Speed Atomic Force Microscopy movie of Snf7 spirals forming during rapid Snf7 polymerization on a polydimethylsiloxane-supported lipid bilayer (PDMS-SLB).

File name: Supplementary Movie 11

Description: High-Speed Atomic Force Microscopy movie of dynamic Snf7 spiral remodeling and densification on a polydimethylsiloxane-supported lipid bilayer (PDMS-SLB).

File name: Supplementary Movie 12

Description: High-Speed Atomic Force Microscopy movie of Snf7/Vps2/Vps24 spirals on a polydimethylsiloxane-supported lipid bilayer (PDMS-SLB) showing different filament packing compared to Snf7-only spirals.

File name: Supplementary Movie 13

Description: High-Speed Atomic Force Microscopy movie of individual Snf7 spiral on a polydimethylsiloxane-supported lipid bilayer (PDMS-SLB) with the inner turn of the spiral undergoing out-of-plane transition, with time averages of frames before and after transition.

File name: Supplementary Movie 14

Description: High-Speed Atomic Force Microscopy movie of individual Snf7 spiral on a polydimethylsiloxane-supported lipid bilayer (PDMS-SLB) with the inner turn of the spiral undergoing out-of-plane transition, with time averages of frames before and after transition.

File name: Supplementary Movie 15

Description: High-Speed Atomic Force Microscopy movie of individual Snf7 spiral on a polydimethylsiloxane-supported lipid bilayer (PDMS-SLB) with the inner turn of the spiral undergoing out-of-plane transition, with time averages of frames before and after transition.

File name: Supplementary Movie 16

Description: High-Speed Atomic Force Microscopy movie of individual Snf7 spiral on a polydimethylsiloxane-supported lipid bilayer (PDMS-SLB) with the inner turn of the spiral undergoing reversible out-of-plane transitions, with time averages of frames before and after transition.

File name: Supplementary Movie 17

Description: High-Speed Atomic Force Microscopy movie of individual Snf7/Vps24/Vps2 spiral on a polydimethylsiloxane-supported lipid bilayer (PDMS-SLB) with the inner turn of the spiral undergoing out-of-plane transitions, with time averages of frames before and after transition.
