# Supplementary material for: Snf7 spirals sense and alter membrane curvature
Source: Nat Commun. 2022 Apr 21;13:2174. doi: 10.1038/s41467-022-29850-z (PMC9023468; doi:10.1038/s41467-022-29850-z)
Supplement: Supplementary file 20 — Reporting Summary [file 41467_2022_29850_MOESM20_ESM.pdf]

nature portfolio

Corresponding author(s): Simon Scheuring

Last updated by author(s): Feb 28, 2022

Reporting Summary

Nature Portfolio wishes to improve the reproducibility of the work that we publish. This form provides structure for consistency and transparency in reporting. For further information on Nature Portfolio policies, see our Editorial Policies and the Editorial Policy Checklist.

Statistics

For all statistical analyses, confirm that the following items are present in the figure legend, table legend, main text, or Methods section.

n/a	Confirmed	
☐	☒	The exact sample size (*n*) for each experimental group/condition, given as a discrete number and unit of measurement
☐	☒	A statement on whether measurements were taken from distinct samples or whether the same sample was measured repeatedly
☒	☐	The statistical test(s) used AND whether they are one- or two-sided<br>*Only common tests should be described solely by name; describe more complex techniques in the Methods section.*
☒	☐	A description of all covariates tested
☒	☐	A description of any assumptions or corrections, such as tests of normality and adjustment for multiple comparisons
☐	☒	A full description of the statistical parameters including central tendency (e.g. means) or other basic estimates (e.g. regression coefficient) AND variation (e.g. standard deviation) or associated estimates of uncertainty (e.g. confidence intervals)
☒	☐	For null hypothesis testing, the test statistic (e.g. *F*, *t*, *r*) with confidence intervals, effect sizes, degrees of freedom and *P* value noted<br>*Give P values as exact values whenever suitable.*
☒	☐	For Bayesian analysis, information on the choice of priors and Markov chain Monte Carlo settings
☒	☐	For hierarchical and complex designs, identification of the appropriate level for tests and full reporting of outcomes
☒	☐	Estimates of effect sizes (e.g. Cohen's *d*, Pearson's *r*), indicating how they were calculated

Our web collection on statistics for biologists contains articles on many of the points above.

Software and code

Policy information about availability of computer code

Data collection	Igor Pro 7 on HS-AFM (SS-NEX, RIBM, Tsukuba, Japan)
Data analysis	MATLAB R2019b, ImageJ 1.52, custom algorithms (github.com/Nebojsa-Jukic/Snf7_scripts)

For manuscripts utilizing custom algorithms or software that are central to the research but not yet described in published literature, software must be made available to editors and reviewers. We strongly encourage code deposition in a community repository (e.g. GitHub). See the Nature Portfolio guidelines for submitting code & software for further information.

Data

Policy information about availability of data

All manuscripts must include a data availability statement. This statement should provide the following information, where applicable:

- Accession codes, unique identifiers, or web links for publicly available datasets
- A description of any restrictions on data availability
- For clinical datasets or third party data, please ensure that the statement adheres to our policy

All data are available within the article and its supplementary information files and we provided the source data in a .xlsx file. The raw HS-AFM date is saved in a file format named .asd and can only be opened using the RIBM HS-AFM software. The raw data can be requested from the corresponding author in a readable format and will be provided at the earliest convenience. We further upload TIFF stacks of the movies analyzed in Figure 2 and Figure 4 along with the codes on github.

Field-specific reporting

Please select the one below that is the best fit for your research. If you are not sure, read the appropriate sections before making your selection.

☒ Life sciences ☐ Behavioural & social sciences ☐ Ecological, evolutionary & environmental sciences

For a reference copy of the document with all sections, see nature.com/documents/nr-reporting-summary-flat.pdf

Life sciences study design

All studies must disclose on these points even when the disclosure is negative.

Sample size	No sample-size calculation was performed for HS-AFM imaging of Snf7 on either substrate described herein. The actual sample size of HS-AFM images of Snf7 spirals on non-planar surfaces as shown in Figure 3 was increased until the total number of analyzed Snf7 spirals for each condition exceeded n=75 (see Fig. 3 caption). All experiments were performed using at least 3 different biological samples and repeated at least 3 times.
Data exclusions	Snf7 spirals that were only partially within the imaging area were excluded from analysis, because their size and morphology could not be unambiguously determined. No other data was excluded.
Replication	Plasma treatment and repetitive experimental use of non-planar surfaces presented herein introduces slight variations of surface topography between each experiment. Thus the data presented for each condition in Figure 3 represent the results of observations from several imaging areas (see Fig. 3 caption) acquired during single experiments on different days and with different HS-AFM tips. Representative Snf7 spiral buckling events were observed in separate HS-AFM experiments performed on different days.
Randomization	This study did not allocate experimental groups. No randomization was required for the reported experiments.
Blinding	This is a molecular biophysics / structural biology study followed by computational data analysis - no blinding was required for this study. Blinding was not required for the reported experiments, all data were analyzed using the same computational methods.

Reporting for specific materials, systems and methods

We require information from authors about some types of materials, experimental systems and methods used in many studies. Here, indicate whether each material, system or method listed is relevant to your study. If you are not sure if a list item applies to your research, read the appropriate section before selecting a response.

Materials & experimental systems

n/a	Involved in the study
☒	☐ Antibodies
☒	☐ Eukaryotic cell lines
☒	☐ Palaeontology and archaeology
☒	☐ Animals and other organisms
☒	☐ Human research participants
☒	☐ Clinical data
☒	☐ Dual use research of concern

Methods

n/a	Involved in the study
☒	☐ ChIP-seq
☒	☐ Flow cytometry
☒	☐ MRI-based neuroimaging
